# The cytoplasmic PAS_C_ domain of the sensor kinase DcuS of *Escherichia coli*: role in signal transduction, dimer formation, and DctA interaction

**DOI:** 10.1002/mbo3.127

**Published:** 2013-09-09

**Authors:** Christian Monzel, Pia Degreif-Dünnwald, Christina Gröpper, Christian Griesinger, Gottfried Unden

**Affiliations:** 1 Institute for Microbiology and Wine Research, Johannes Gutenberg-UniversityMainz, Germany; 2 Max-Planck-Institute for Biophysical ChemistryGöttingen, Germany

**Keywords:** DctA, DcuS sensor kinase, fumarate, PAS domain, signal transduction.

## Abstract

The cytoplasmic PAS_C_ domain of the fumarate responsive sensor kinase DcuS of *Escherichia coli* links the transmembrane to the kinase domain. PAS_C_ is also required for interaction with the transporter DctA serving as a cosensor of DcuS. Earlier studies suggested that PAS_C_ functions as a hinge and transmits the signal to the kinase. Reorganizing the PAS_C_ dimer interaction and, independently, removal of DctA, converts DcuS to the constitutive ON state (active without fumarate stimulation). ON mutants were categorized with respect to these two biophysical interactions and the functional state of DcuS: type I-ON mutations grossly reorganize the homodimer, and decrease interaction with DctA. Type IIA-ON mutations create the ON state without grossly reorganizing the homodimer, whereas interaction with DctA is decreased. The type IIB-ON mutations were neither in PAS_C_/PAS_C__,_ nor in DctA/DcuS interaction affected, similar to fumarate activated wild-typic DcuS. OFF mutations never affected dimer stability. The ON mutations provide novel mechanistic insight: PAS_C_ dimerization is essential to silence the kinase. Reorganizing the homodimer and its interaction with DctA activate the kinase. The study suggests a novel ON homo-dimer conformation (type IIB) and an OFF conformation for PAS_C_. Type IIB-ON corresponds to the fumarate induced wild-type conformation, representing an interesting target for structural biology.

## Introduction

**Figure 1:**
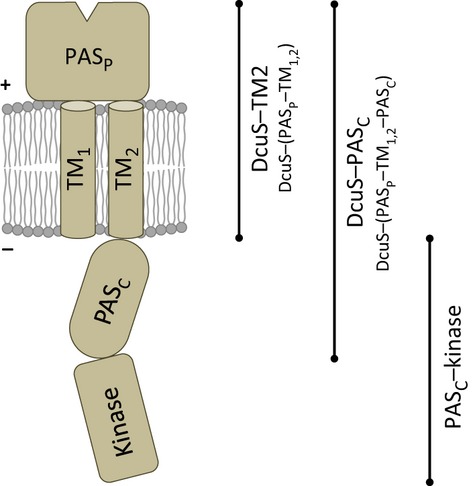
Schematic presentation of the DcuS domains PAS_P_, TM1, 2, PAS_C_, and kinase. Various constructs used here and their domain composition are shown with the corresponding designation, and the terminology used by Etzkorn et al. (2008) (small lettering). For simplicity DcuS is shown only as monomer and domain connecting linker regions are omitted; the dimeric structure of DcuS is presented in Figure 10.

The sensor kinase DcuS is part of the fumarate (or C_4_-dicarboxylate) responsive two-component system DcuS/DcuR of *Escherichia coli* (Zientz et al. 1998; Scheu et al. 2010a). DcuS makes up together with the citrate sensor kinase CitA, the CitA family of sensor kinases. DcuS is membrane integral and represents an extracytoplasmic (or periplasmic) sensing histidine kinase (Mascher et al. 2006). It contains an input or sensory domain that is accessible from the extracellular space, and a cytoplasmic portion consisting of a PAS (PAS_C_) and a phosphotransfer (or kinase) domain (Fig. 1). The sensory and the cytoplasmic regions are linked by a transmembrane region that consists of the transmembrane helices TM1 and TM2 which are important for the signal transfer across the membrane (Scheu et al. 2010a). DcuS is a functional homodimer in the presence or the absence of an effector like fumarate (Scheu et al. 2012). DcuS forms a sensor complex with the succinate transporter DctA during aerobic growth which is required for normal response to fumarate or succinate (Witan et al. 2012a,b). Under anaerobic conditions, the fumarate/succinate antiporter DcuB takes over the function of DctA as cosensor of DcuS (Kleefeld et al. 2009). In the absence of substrates like fumarate, the idle transporters inhibit DcuS function, whereas transport-active DctA or DcuB allow transfer of DcuS to the active state and autophosphorylation (Witan et al. 2012b). When DctA or DcuB are missing in deletion strains, DcuS exists in the permanent active state and requires no further C_4_-dicarboxylate binding for activation (Davies et al. 1999; Kleefeld et al. 2009; Witan et al. 2012a). It has been suggested that transport-active DctA interacts differently if at all with the PAS_C_ domain of DcuS which switches then to the ON state resulting in active kinase (Witan et al. 2012b).

The input or sensory domains of DcuS and of the related CitA are PAS (Per-ARNT-SIM) domains (Pappalardo et al. 2003; Reinelt et al. 2003; Cheung and Hendrickson 2008; for reviews see Scheu et al. 2010a; Kneuper et al. 2010) also called PDC domains (Cheung and Hendrickson 2008). In CitA of *Klebsiella pneumoniae* binding of citrate causes compaction of the PAS_P_ domain which is supposed to pull the TM2 region in the direction of PAS_P_ (Sevvana et al. 2008), and to transfer the signal across the membrane and to PAS_C_. The same conformational change can be inferred from the NMR structure (Pappalardo et al. 2003) and the X-ray structure (Cheung and Hendrickson 2008) of DcuS in response to C_4_-dicarboxylate binding (Scheu et al. 2010a), resulting in signal transfer by PAS_C_. The kinase domain is phosphorylated at the conserved His349 residue (Janausch et al. 2002), and as for other sensor histidine kinases the phosphorylation within the DcuS dimer is believed to come about in trans (Stock et al. 2000).

PAS_C_ of DcuS has been shown to function in signal transfer from the membrane to the kinase (Etzkorn et al. 2008) and as a signal input site from the cosensors DctA and DcuB (Witan et al. 2012a,b). Molecular details of both functions are not clear, but structural and mutational studies imply that control of protein interaction in the PAS_C_ dimer plays an important role (Etzkorn et al. 2008) as suggested for other PAS domain functions (Möglich et al. 2009, 2010). PAS_C_ is a homodimer and has a fold similar to other cytoplasmic PAS domains with a five-stranded antiparallel β-sheet and surrounding α-helices (Etzkorn et al. 2008; Kneuper et al. 2010; Scheu et al. 2010b, 2012). In a membrane embedded construct point mutations of the PAS_C_ domain were obtained that inferred permanent ON or OFF state to DcuS. ON mutations in residues Asn248 and Asn304 caused permanent (fumarate independent) activation of the kinase. The effect of the mutation was suggested by a potential decrease in dimer interaction due to the loss of Asn248/Asn248, and possibly also of Asn304/Lys232 contacts in the PAS_C_ homodimer. Change in the dimerization was supposed to transmit a stimulating signal to the kinase domain (Etzkorn et al. 2008) in simulation of structural changes induced by binding of the effector fumarate to PAS_P_ under physiological conditions.

Here, we set out to study PAS_C_ of DcuS and its role in controlling the function of the kinase domain in vivo on the basis of the suggestions of Etzkorn et al. (2008) and Witan et al. (2012b) that signal transmission from PAS_C_ to the kinase involves changes in the dimer stability of PAS_C_, and that the cosensor DctA affects the functional state of PAS_C_ by direct interaction. To this end ON and OFF variants of PAS_C_ were identified. Both ON and OFF phenotypes were characterized by relating the site of mutation to changes in (i) the functional state of DcuS, (ii) the homodimerization of DcuS, and (iii) the interaction of DcuS with DctA. These aspects provide information on the supposed link between PAS_C_ dimer interaction, control of kinase activity, and signal perception from DctA. The functional state of DcuS was tested by measuring expression of the DcuS-DcuR dependent reporter gene fusion *dcuB-lacZ*. Interaction of PAS_C_ in the DcuS homodimer, or DcuS-DctA interaction, was characterized in vivo by the use of a bacterial two-hybrid system and by chemical cross-linking.

## Methods

### Bacteria and molecular genetics methods

Derivatives of *E. coli* K12 and plasmids used are listed in Tables 1 and S1. All molecular methods were performed according to standard procedures (Sambrook and Russel 2001). Plasmids were isolated using the GeneJET™ Plasmid Miniprep Kit and PCR products were purified using the GeneJET™ PCR Purification Kit (Fermentas, St. Leon-Rot, Germany). Oligonucleotides were synthesized by Sigma-Aldrich (St. Louis, MO) or Eurofins MWG Operon (Ebersberg, Germany). Transformation of *E. coli* was accomplished through electroporation (Dower et al. 1988) or heat shock. Point mutations in plasmids were created with *Pfu* DNA Polymerase (Fermentas, St. Leon-Rot, Germany) in combination with *Dpn*I endonuclease (Fermentas, St. Leon-Rot, Germany) and heat shock-competent XL1-Blue (Agilent Technologies, Santa Clara, CA). Antibiotics were used at the following concentrations: 100 μg/mL ampicillin, 20 μg/mL chloramphenicol, 50 μg/mL kanamycin, 50 μg/mL spectinomycin, 50 μg/mL streptomycin, 15 μg/mL tetracycline. Concentration was halved if two or more antibiotics were used simultaneously.

**Table 1:** Strains of *Escherichia coli* and plasmids used in this study

Strain or plasmid	Genotype	Reference or source
*Escherichia coli* K-12		
MC4100	F^−^ *araD139* Δ(*argF-lac*)*U169 rpsL150* Δ*lacZ relA1 flbB530 deoC1 ptsF25 rbsR*	Silhavy et al. (1984)
JM109	*recA1 supE44 endA1 hsdR17 gyrA96 relA1 thi* Δ(*lac-proAB*) F'[*traD36 proAB*^+^, *lacI*^q^ *lacZ*ΔM15]	Yanisch-Perron et al. (1985)
IMW260	MC4100 λ[Φ(*dcuB'-'lacZ*)*hyb, bla*^*+*^] *dcuS*::Cam^r^	Zientz et al. (1998)
IMW536	MC4100 λ[Φ(*dcuB'-'lacZ*)*hyb, bla*^*+*^] *dcuS*::Cam^r^, dcuB::Spec^r^	Kleefeld et al. (2009)
BTH101	F^−^ *cya-99*, *araD139*, *galE15*, *galK16*, *rpsL1* (*Strr*), *hsdR2*, *mcrA1*, *mcrB1*	Karimova et al. (2005)
Plasmids for reporter gene measurements		
pET28a	Expression vector, pBR *ori*, T7 Promoter, His-tag (Kan^r^)	Novagen
pME6010	Cloning vector, pVS1 shuttle vector (Tet^r^)	Heeb et al. (2000)
pMW181	pET28a with *dcuS* (2.2 kb *Xba*I/*Hind*III fragment) (Kan^r^)	Kneuper et al. (2005)
pMW228	pME6010, but with complete *dcuB* gene with own promoter (Tet^r^)	Kim et al. (2007)
Plasmids for BACTH measurements		
pUT18	N-terminal T18 protein fusion plasmid, pUC19 derivative (Amp^r^)	Karimova et al. (2005)
pUT18C	C-terminal T18 protein fusion plasmid, pUC19 derivative (Amp^r^)	Karimova et al. (2005)
pKNT25	N-terminal T25 protein fusion plasmid, pSU40 derivative (Kan^r^)	Karimova et al. (2005)
pKT25	C-terminal T25 protein fusion plasmid, pSU40 derivative (Kan^r^)	Karimova et al. (2005)
pUT18C-zip	T18-Zip expression plasmid, pUT18C derivative (Amp^r^)	Karimova et al. (2005)
pKT25-zip	T25-Zip expression plasmid, pKNT25 derivative (Kan^r^)	Karimova et al. (2005)
pMW426	T25-DcuS expression plasmid, pKT25 derivative (Kan^r^)	Scheu et al. (2012)
pMW429	T18-DcuS expression plasmid, pUT18C derivative (Amp^r^)	Scheu et al. (2012)
pMW856	T25-DctA expression plasmid, pKT25 derivative (Kan^r^)	This study
pMW948	DcuS-TM2-T18 expression plasmid (DcuS(1-206)), pUT18 derivative (Amp^r^)	This study
pMW949	DcuS-TM2-T25 expression plasmid (DcuS(1-206)), pKNT25 derivative (Kan^r^)	This study
pMW950	DcuS-PAS_C_-T18 expression plasmid (DcuS(1-326)), pUT18 derivative (Amp^r^)	This study
pMW951	DcuS-PAS_C_-T25 expression plasmid (DcuS(1-326)), pKNT25 derivative (Kan^r^)	This study
pMW952	PAS_C_-T18 expression plasmid (DcuS(211-326)), pUT18 derivative (Amp^r^)	This study
pMW953	PAS_C_-T25 expression plasmid (DcuS(211-326)), pKNT25 derivative (Kan^r^)	This study
pMW954	Kinase-T18 expression plasmid (DcuS(330-539)), pUT18 derivative (Amp^r^)	This study
pMW955	Kinase-T25 expression plasmid (DcuS(330-539)), pKNT25 derivative (Kan^r^)	This study
pMW1075	PAS_C_-kinase-T25 expression plasmid (DcuS(211-539)), pKNT25 derivative (Kan^r^)	This study
pMW1076	PAS_C_-kinase-T18 expression plasmid (DcuS(211-539)), pUT18 derivative (Amp^r^)	This study
pMW1126	DctA_400-428_-T25 expression plasmid, pKNT25 derivative (Kan^r^)	This study
pMW1416	His6-pUT18. pUT18 encoding fusion proteins with an N-terminal 6xHis-tag	This study
pMW1417	His6-pKNT25. pKNT25 encoding fusion proteins with an N-terminal 6xHis-tag	This study
pMW1656	His6-PAS_C_-T18 expression plasmid (DcuS(211-326)), pMW1416 derivative (Amp^r^)	This study
pMW1657	His6-PAS_C_-T25 expression plasmid (DcuS(211-326)), pMW1417 derivative (Kan^r^)	This study
pMW1658	His6-Kinase-T18 expression plasmid (DcuS(211-539)), pMW1416 derivative (Amp^r^)	This study
pMW1659	His6-Kinase-T25 expression plasmid (DcuS(211-539)), pMW1417 derivative (Kan^r^)	This study
pMW1911	T18-PAS_C_ expression plasmid, pUT18C derivative (Amp^r^)	This study

The table gives only a basic list of plasmids and constructs, the complete list can be found in Table S1.

### Random mutagenesis using error-prone PCR

A random library of single, double, and multiple mutants was generated using the error-prone PCR method of Cadwell and Joyce (1992). A fragment of *dcuS* encoding PAS_C_ was amplified from plasmid pMW181 with the primer pairs dcuS-pMW440-for (5′-CAT GCT GGT CGG ACT GAT TGG-3′) and dcuS-pMW437/439/440-rev(5′-GAC CAG ACC GTC GAG TCG CTG-3′). PCR was performed with *Taq* polymerase under increased MgCl_2_/MnCl_2_ concentration and a large number of cycles. Using the restriction sites of *Bve*I and *Sna*BI the amplified region of *dcuS* was replaced by the PCR product to get full-length *dcuS* with intact reading-frame. The mixture was transformed into *E. coli* to achieve single clones and these clones were then sequenced for point mutation(s) within *dcuS*.

### Construction of plasmids

For deletion of the PAS_C_ domain within full-length DcuS *Eco*RI restriction sites were introduced into pMW181 at both ends of PAS_C_ using the following primers: PAS_C_-EX1-for (5′-CTG AAA AAA ATC GAA TTC GGC CTG GAA CCC-3′) and PAS_C_-EX1-rev (5′-GGG TTC CAG GCC GAA TTC GAT TTT TTT CAG-3′), PAS_C_-EX5-for (5′-CAA CCT TCA GGG ACG AAT TCG AAG TAC G-3′) and PAS_C_-EX5-rev (5′-CGT ACT TCG AAT TCG TCC CTG AAG GTT G-3′). Digestion with *Eco*RI and subsequent ligation of the cut vector resulted in the DcuS-ΔPAS_C_ construct (pMW1168).

For protein interaction studies with the bacterial two-hybrid system (BACTH) (Karimova et al. 1998) full-length DcuS, truncated DcuS, or isolated domains of DcuS were fused to the T18 and T25 domains. N-terminally fused full-length T18-DcuS (pMW429) and T25-DcuS (pMW426) were constructed as described previously (Scheu et al. 2012). C-terminally fused T18- and T25-constructs of truncated DcuS were obtained by amplifying parts of *dcuS* from pMW181 with the primer pairs THS_dcuS-f (5′-CAC ACA AGG ATC CGA TGA GAC ATT CAT TGC-3′) plus THS_dcuS-PAS_C_-r (5′-CGC TGC ATC AGT TTA CGT GAA TTC GTT TTG TC-3′) for DcuS-PAS_C_ (pMW950; pMW951) and THS_dcuS-TM2-r (5′-GCC GAA AAG GAT TGA ATT CAG TAC CTT AAC CAG-3′) for DcuS-TM2 (pMW948; pMW949), respectively, and subsequent cloning into pUT18 and pKNT25. The isolated PAS_C_-kinase was fused to T18 and T25 at their C-terminus (pMW1076, pMW1075) by amplifying those domains from pMW181 with the primer pairs THS_PAS_C_-f (5′-CTT TTG GAT CCG GAA CCC TAC-3′) plus kinase-r (5′-CGA TAA TTA ATA CAT GAA TTC CTG TTC G-3′) followed by cloning into pUT18 and pKNT25. Fusions of the positive control, ZiP-T18, and ZiP-T25, were obtained as part of the BACTH system.

### β-galactosidase assay

The *dcuB-lacZ* expression was measured as the β-galactosidase activity within the exponential growth phase (ΔOD_578_ 0.5–0.8). Cells were grown in 96 deep well plates, anaerobically at 37°C under an atmosphere of N_2_ in enriched mineral (eM9) medium supplemented with acid-hydrolyzed casamino acids (0.1%), l-tryptophan (0.005 %), glycerol (50 mmol/L), and dimethyl sulfoxide (DMSO) (20 mmol/L). Sodium fumarate (20 mmol/L) was used as indicated. The activities were quantified according to Miller (1992) in 96-well microtiter plates, at least in triplicate for each experiment. Optical density at 570 nm and extinction at 415 nm were measured with a volume of 315 μL per well. Cell permeabilization was achieved for 200 μL cell culture in 800 μL of a 0.1 mol/L potassium phosphate buffer supplemented with 10 mmol/L potassium chloride, 1 mmol/L magnesium chloride, 0.005% (w/v) cetyltrimethylammonium bromide (CTAB), 0.0025% (w/v) sodium deoxycholate, and 0.0027% (v/v) 2-mercaptoethanol. A volume of 215 μL permeabilized cell culture was incubated with 40 μL 0.4% (w/v) *ortho*-nitrophenyl-β-galactoside (ONPG) at 30°C. The reaction was stopped after 20 min with 60 μL 1 mol/L sodium carbonate.

For measuring the protein interaction in the BACTH system *E. coli* BTH101 was cotransformed with pairs of plasmids encoding T18 and T25 fusions and grown on Luria-Bertani (LB) agar plates for 40 h at 30°C. Liquid cultures were conducted in 48-well plates with 500 μL LB medium per well at 30°C and vigorous shaking. Cells were grown to the exponential growth phase (ΔOD_578_ 0.5–0.8) and the β-galactosidase was measured as described above. Where indicated, the cell growth was altered in the following way: *E. coli* BTH101 was cotransformed with the T18- and T25-fusion plasmids and grown for 66 h on LB agar plates without a chromogenic substrate like X-Gal. For the β-galactosidase assay colonies were resuspended in an appropriated amount of fresh LB medium to obtain a “liquid culture” with an ΔOD_578_ comparable to cells in the exponential phase.

### In vivo cross-linking

A cysteine-free variant of DcuS on an arabinose-inducible expression vector (pMW967) (Scheu et al. 2008) and variants carrying the mutations N248D, V276A, or N304D were transformed into *E. coli* JM109. Cells were grown at 30°C in LB medium supplemented with 300 μmol/L l-arabinose as the inducer. Exponential-phase cells were harvested, washed with phosphate buffered saline (PBS) buffer, pH 7.5, and resuspended in PBS buffer. To start cross-linking disuccinimidyl suberate (DSS), dissolved at a concentration of 25 mmol/L in DMSO, was added to the cells at a final concentration of 30 μmol/L. After incubation for 15 min at 20°C the reaction was stopped by the addition of Tris-HCl, pH 7.7 at a final concentration of 100 mmol/L. The mixture was subjected to SDS-PAGE (sodium dodecyl sulfate polyacrylamide gel electrophoresis), Western-blot, and immunostaining.

### SDS-PAGE, Western-blot, and immunostaining

The control for expression of noninteracting fusion proteins was done with cells growing under conditions comparable to the respective experiment. Cells were harvested and diluted in PBS, pH 7.5, to a final protein concentration of 10 μg/mL (OD 1 at 578 nm corresponding to 280 μg protein mL^−1^). Samples were dissolved and boiled (95°C, 5 min) in 2× SDS sample buffer containing 200 mmol/L dithiothreitol (Laemmli 1970), subjected to SDS-PAGE (stacking gel: 4%; resolving gel: 10%) and transferred to a nitrocellulose membrane (Towbin et al. 1979). For immunostaining rabbit polyclonal antiserum raised against the periplasmic domain of DcuS (Eurogentec, Seraing, Belgium) and rabbit polyclonal purified antibody against His-tagged proteins (Carl Roth, Karlsruhe, Germany), respectively, were used. Primary antibodies were detected with secondary anti-rabbit IgG antibodies coupled to peroxidase (Sigma Aldrich, St. Louis, MO).

## Results

### PAS_C_ silences the kinase of DcuS

**Figure 2:**
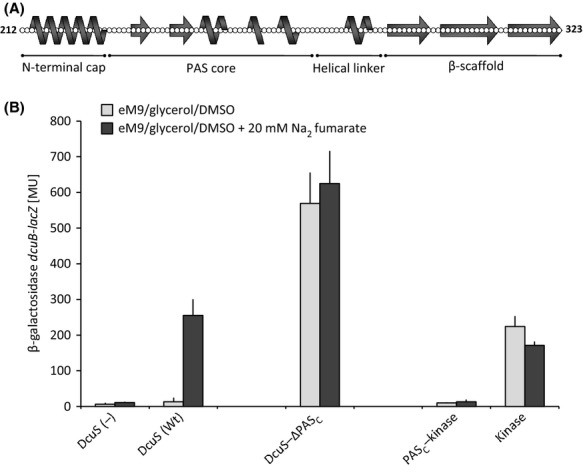
Expression of *dcuB-lacZ* in *Escherichia coli* IMW260 (*dcuS*) containing *dcuS* with a deleted PAS_C_ domain. (A) The subdomains of PAS_C_ with the α-helices and β-sheets according to Etzkorn et al. (2008). (B) The expression of *dcuB-lacZ* in the *dcuS* inactive strain IMW260 that was supplemented with a plasmid encoding full-length DcuS (DcuS_Wt_) or DcuS with full deletion of PAS_C_. The right side shows the activity of cytosolic fragments of DcuS, comprising the PAS_C_-kinase or the kinase constructs.

PAS_C_ consists of PAS-characteristic subdomains (Etzkorn et al. 2008) including the N-terminal cap, the PAS core, the helical linker, and the β-scaffold (Fig. 2A). The complete PAS_C_ domain or subdomains of it were removed in internal deletions from a plasmid encoding *dcuS*. The variants were tested by in vivo complementation of a *dcuS-*negative strain. Deletion of PAS_C_ (Fig. 2B) or subdomains of it (not shown) resulted in high expression of the DcuS-dependent reporter gene, which was constitutive and required no stimulation by fumarate.

A soluble construct comprising only the cytoplasmic parts of DcuS (PAS_C_-kinase, see Fig. 1) was constitutively OFF, that is, it was not able to stimulate expression of *dcuB-lacZ* (Fig. 2B) despite its expression at sufficient yields (Fig. 5B) and the lack of interaction with DctA which is normally required to silence full-length DcuS (Fig. 6C). In contrast, production of the kinase domain without PAS_C_ resulted in an ON variant, that is, substantial *dcuB-lacZ* expression (87% of fumarate stimulated wild-typic DcuS), which was fumarate independent. It can be concluded that the PAS_C_ domain without the transmembrane and periplasmic domain of DcuS inhibits the kinase and autophosphorylation even in the absence of DctA. In full-length DcuS DctA is required to silence the kinase but either addition of fumarate or removal of DctA relieve the kinase inhibition by PAS_C_ (Witan et al. 2012a).

### ON mutations in PAS_C_ with fumarate independent expression of *dcuB-lacZ*

In a previous study, two variants of PAS_C_ (DcuS-N248A and DcuS-N304D) had been isolated in the supposed PAS_C_ homodimerization site (Etzkorn et al. 2008) and were found to be ON-mutations. To test the hypothesis that the location of the mutations is related to dimer interaction and the functional state, PAS_C_ was screened by directed and random mutagenesis for ON and OFF mutations. Residues for directed mutagenesis were spotted by sequence comparison with homologous signal transducing PAS domains of PAS2 of NifL from *Azotobacter vinelandii* (Slavny et al. 2010) and of Aer from *E. coli* (Repik et al. 2000; Campbell et al. 2010).

**Figure 3:**
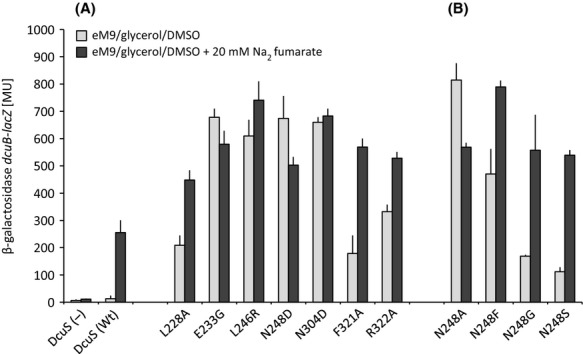
ON variants of PAS_C_ as measured in full-length DcuS. The figure gives an overview over selected characteristic ON mutations in PAS_C_ that exceed in the noninduced state (gray bar) the induction level of 60 Miller units (corresponding to 10-fold noninduced level of wild type) in eM9 medium with glycerol plus DMSO under anaerobic conditions. Black bars show induction after anaerobic growth in the same medium with 20 mmol/L sodium fumarate. The expression was tested in strain IMW260 (*dcuS* negative) after complementation with plasmid encoded variants of DcuS (derivatives of pMW181). In addition to the ON mutations shown here, also substitutions F221I, M227V/L, L228D, E233D, V235D, V236D, A237D, I247D, N248A/G/S, D291A/N, V308D, I315A, A317D, I318D, S319P, and T320A showed a fumarate-independent *dcuB-lacZ* expression. See Tables 1 and S1 for the corresponding plasmids.

The variants of DcuS obtained by random mutagenesis were tested in the *dcuS*-less strain IMW260 with the *dcuB-lacZ* reporter fusion. ON mutants showed expression of *dcuB-lacZ* already in the absence of fumarate, whereas in OFF mutants expression of *dcuB-lacZ* was missing even in the presence of fumarate. By directed and random mutagenesis altogether 65 (58%) of the 112 amino acid residues of PAS_C_ were mutated, yielding ON mutations at 20 sites with 30 different variants, and OFF mutations at seven different amino acid positions. A selection of the ON mutations is given in Figure 3, a complete survey in Figure S4. The mutations at 44 sites were silent (50% to 200% of wild-typic activity of *dcuB-lacZ* expression), but six of the silent mutations were ON or OFF mutants depending on the type of replacements.

Sites for ON mutations were located mainly in the PAS core and the β-scaffold region with a clustering at the C- and N-terminal parts, respectively, which contain also the mutations described in Etzkorn et al. (2008). Testing of the membrane-embedded variant of DcuS with mutation N248D by solid state NMR (Etzkorn et al. 2008) gave no indication for gross structural changes in PAS_C_ (not shown). Mutations where *dcuB-lacZ* expression exceeded in the noninduced state that of the wild type by a factor of 10 (60 vs. 6 Miller units) were termed “ON” mutants. The reporter protein expression in the induced state was generally higher than for wild-typic DcuS. While some ON mutants where still fumarate sensible (e.g., L228A, F321A, R322A), in other ON mutants the noninduced reporter expression level was close to that of the fumarate induced state (e.g., L246R, N248D), or fumarate caused even a slight repression (e.g., E233G, N248D) (Fig. 3A). ON mutations at positions L246 and N248 were at sites corresponding to essential residues in PAS2 of NifL_Av_ (Slavny et al. 2010).

The kind of substitution was functionally crucial for most ON mutants (Fig. 3B). Mutations at position N248 caused a constitutive ON state when the residue was replaced by Asp, Ala, and (in parts also) by a Gly residue, whereas replacement by Phe and Ser produced partial or weak ON mutants. Similar observations were obtained for other residues (not shown). Introducing charged residues at neutral or hydrophobic positions produced often a constitutive ON phenotype (e.g., L246R, I318D, or A237D), whereas the replacement by a hydrophobic or less polar residue was silent (e.g., L246A, I318A, or A237C).

**Figure 4:**
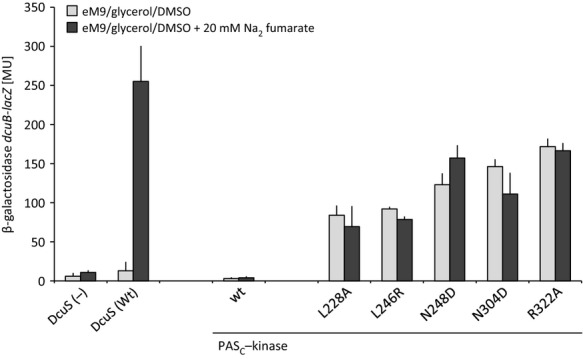
ON variants of PAS_C_ as measured in the cytosolic PAS_C_-kinase construct. The figure shows the expression of *dcuB-lacZ* induced by the presence of cytosolic PAS_C_-kinase of DcuS in the wild-typic form and after introduction of ON mutations, respectively. Gray and black bars: induction after anaerobic growth in eM9 with glycerol plus DMSO without or with 20 mmol/L sodium fumarate, respectively. The expression was tested in strain IMW260 (deficient for *dcuS*) after complementation with plasmid-encoded variants of PAS_C_-kinase-T25 of DcuS (plasmid pMW1075, and variants of it). See Tables 1 and S1 for the corresponding plasmids. The activity is compared to expression in IMW260 and IMW260 complemented with DcuS (pMW181; “DcuS(Wt)”).

As shown in Figure 2, PAS_C_ inhibits the kinase activity also in the soluble PAS_C_-kinase construct. For testing whether the ON mutations reflect a PAS_C_ intrinsic property, the mutations were introduced into the PAS_C_-kinase (see Fig. 1) construct and tested for their capacity to stimulate expression of *dcuB-lacZ* (Fig. 4). Whereas wild-typic PAS_C_-kinase protein was not able to induce expression of *dcuB-lacZ*, each of the ON variants showed high fumarate-independent expression of *dcuB-lacZ*. Therefore, the ON mutations are PAS_C_ intrinsic traits and independent of PAS_P_ or other regions of DcuS.

### Effect of mutations in PAS_C_ and of fumarate on dimerization of DcuS

DcuS is a functional homodimer or homo-oligomer (Scheu et al. 2012). Structural studies suggested that dimer stability in the PAS_C_ domain might be decreased in ON mutants (Etzkorn et al. 2008), and it was suggested that the decreased stability is related to activation of DcuS kinase. Experiments were performed to test in vivo whether the dimer interaction of PAS_C_ or DcuS is changed in PAS_C_ ON mutations. The interaction was tested by use of the BACTH system which allows in situ probing of protein interaction. The bacterial two-hybrid system (Karimova et al. 1998) has been shown earlier by comparison with FRET studies to provide reliable data on DcuS interaction (Scheu et al. 2010a, 2012; Witan et al. 2012b). The T18 and T25 reporter fusions for testing conformational changes were located at the C- or N-terminal ends of DcuS.

**Figure 5:**
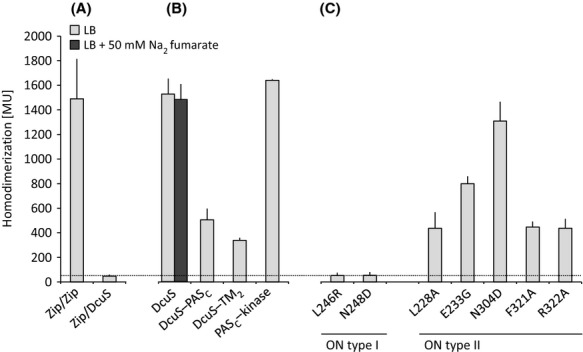
Homodimerization of DcuS (full-length or truncated and wild-typic or PAS_C_ ON and OFF mutants): studies using the bacterial two-hybrid system (BACTH). *Escherichia coli* BTH101 was cotransformed with pairs of plasmids (T18 and T25) encoding: (A) leucine zippers as a positive control for interaction or a leucine zipper plus full-length DcuS as the respective negative control for background β-galactosidase activity. (B) N-terminally fused full-length DcuS or C-terminally fused DcuS-PAS_C_, DcuS-TM2, or PAS_C_-kinase. (C) Variants of N-terminally fused full-length DcuS with the ON mutations shown in the figure. The corresponding plasmids are derivatives of pMW429 and pMW426 (T18-DcuS and T25-DcuS, respectively) that are given in Tables 1 and S1. β-galactosidase activity was measured in LB medium with (black bars) or without (gray bars) 50 mmol/L sodium fumarate. The dotted line represents the background β-galactosidase activity, exceeding this threshold was considered as interaction between the corresponding T18 and T25 fusion proteins.

A positive reaction in the BACTH system depends on the reconstitution of adenylate cyclase activity from the separate T18 and T25 domains of the enzyme that are fused to interacting proteins. Leucine zippers fused to T18 and T25 can be used as a positive control for strong interaction (Fig. 5A), while a combination of two noninteracting proteins like a leucine zipper and full-length DcuS determine the background β-galactosidase activity (Fig. 5A and dotted line in Fig. 5A–C). The reporter strain with plasmids that encode the N-terminal fusions T25-DcuS and T18-DcuS produced high activity of β-galactosidase (Fig. 5B), comparable to the positive control. The high activity suggests efficient interaction of the DcuS monomers (Scheu et al. 2012). C-terminal fusions of full-length DcuS to T18 or T25 were instable (blue colonies but a negative response in the β-galactosidase assay) and therefore not used. Deletion of the kinase and of the kinase plus PAS_C_ domains resulted in decrease of BACTH activity, but the activity exceeded the negative control level significantly (factors 6.1 or higher) indicating homodimerization (Fig. 5B). The dimer interaction of the DcuS-TM2 construct is most probably due to the transmembrane regions as the PAS_P_ domain has little tendency to form dimers (Pappalardo et al. 2003).

The soluble construct, consisting of the cytoplasmic PAS_C_-kinase domains (see Fig. 1), exhibited very high activity and interaction that exceeded even the positive control (Fig. 5B). The strains producing only the isolated domains PAS_C_ or kinase, showed only background level activity (not shown). As the corresponding proteins fused to the T18 and T25 domains were present in the cells (see Fig. S1), the missing response was apparently due to lack of interaction. The kinase is composed of the catalytic and the dimerization domain DHp, and therefore the lack of interaction in this construct is probably artificial and can be caused by interference of the tags with kinase folding. Overall, full-length DcuS, the truncated constructs DcuS-TM2, DcuS-PAS_C_, and the soluble PAS_C_-kinase (compare Fig. 1) form homodimers in vivo.

### Effect of mutations in PAS_C_ on DcuS dimer interaction

ON mutations of PAS_C_ were tested for their effect on DcuS dimer interaction using the BACTH assay. The ON mutations were tested in full-length DcuS and compared to wild-typic DcuS. For wild-typic DcuS the BACTH read out was the same in the fumarate deficient and the fumarate activated state (Fig. 5B). For the ON mutations, two different types of responses were observed. Type I ON mutations like N248D dropped in the BACTH assay to or close to background levels, suggesting that the corresponding mutations affected in the mode of homodimerization or dimer interaction of DcuS (Fig. 5C). We interpret this as a gross reorganization of the PAS_C_ homodimer. These ON mutations showed the same loss of response in the BACTH assay when they were tested in the cytosolic PAS_C_-kinase and the truncated DcuS-PAS_C_ constructs (not shown). This response is compatible with the model of Etzkorn et al. (2008) for DcuS function which predicts that signal transfer to the kinase domain is based on weakening or changes in the dimer interaction of the PAS_C_ homodimer. Here, a similar response as that predicted for mutation N248D was also observed for the other ON mutations of type I.

However, type II ON mutations retained high or significant interaction (or β-galactosidase activity) in the BACTH assay (Fig. 5C) thus implementing an ON state without the gross reorganization characteristic of the type I ON mutations. In particular mutation N304D (the second ON mutation described by Etzkorn et al. 2008) retained high activities in the BACTH assay of the same range as for the fumarate activated wild type. The same applied to the respective PAS_C_-kinase and the DcuS-PAS_C_ constructs that were tested in the BACTH assay (not shown).

To ensure that the changed dimer interaction in the BACTH assay is not due to a lack of production or stability of the corresponding variants, their presence was tested by Western blotting with antisera directed against PAS_P_ of DcuS. Figure S1 demonstrates that the variants were produced at significant levels, so the loss of interaction is not due to lack of the corresponding proteins.

Earlier it has been shown that DcuS can be cross-linked in the membrane of the bacteria by the homo-bifunctional cross-linking reagent disuccinimidyl-suberate (DSS) (Scheu et al. 2010b) that reacts with free amino groups of Lys residues. For the test a Cys-free variant of DcuS was used that does not show artificial cross-linking by disulfide formation. Under these conditions DcuS was present after DSS-treatment and SDS-PAGE in immunoblots as monomeric, dimeric, and tetrameric protein (with the monomer as the major form as described before Scheu et al. 2012). The same experiment was carried out for the ON variant DcuS(N248D). In contrast to the negative response in the BACTH assay (see Fig. 5C), DcuS(N248D) was not affected in the contents of the dimeric and tetrameric forms. Therefore, the decrease in the BACTH response might be linked to conformational changes that interfere with the T18/T25 interaction rather than with monomerization of DcuS. It appears therefore that DcuS is retained as a dimer even in the type I ON mutations where a homodimer reorganization may occur. Maintaining the dimeric state is consistent with the active state of the kinase that is supposed to transphosphorylate in the dimeric state. It has to be taken in mind that the T18 and T25 tags are fused to the C- or N-termini of the DcuS constructs, and therefore the change in BACTH activity reflects conformational change at these sites rather than monomerization which can, however, occur locally.

The physiological stimulus for transferring DcuS into the active state is the presence of external fumarate or of other C_4_-dicarboxylates. When the effect of fumarate on the dimeric interaction was tested by the BACTH assay with full-length DcuS, the high degree of dimerization of DcuS was not affected by the presence of fumarate (Fig. 5B). All other constructs and variants behaved similar with no differences between fumarate presence and absence. This demonstrates that the effect of fumarate on the dimerization of full-length DcuS resembles in this respect that of type II ON mutations by inducing an active state without the gross reorganization of the homodimeric state as in type I ON mutants.

### Loss of DctA/DcuS interaction in PAS_C_-ON mutants

**Figure 6:**
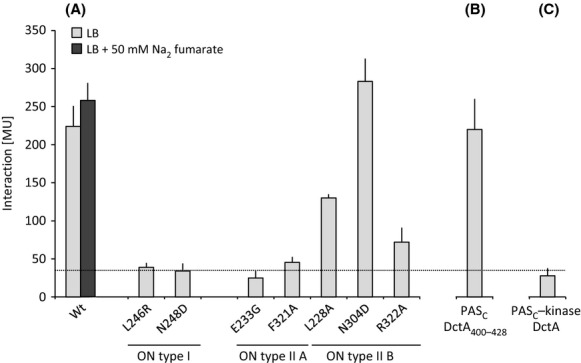
DctA/DcuS interaction in PAS_C_ ON mutations of type I and II. *Escherichia coli* BTH101 was cotransformed with pairs of plasmids encoding: (A) T18-DcuS and T25-DctA (pMW856). Variants of DcuS were used with the ON mutations shown in the figure. The corresponding plasmids are derivatives of pMW429 that are given in Tables 1 and S1. (B) T18-PAS_C_ (pMW1911) and DctA_400-428_-T25 (pMW1126). (C) PAS_C_-kinase-T18 (pMW1076) and DctA-T25 (pMW858). β-galactosidase activity was measured in LB medium with (black bars) or without (gray bars) 50 mmol/L sodium fumarate. The dotted line represents the background β-galactosidase activity, exceeding this threshold was considered as interaction between the corresponding T18 and T25 fusion proteins.

Transport inactive DctA directly interacts with PAS_C_ and forms a DctA/DcuS sensory complex which keeps DcuS in the inactive state (Witan et al. 2012a,b). With transport active DctA the interaction is changed or weakened which allows PAS_C_ to acquire the functional ON state with active kinase (Witan et al. 2012a,b). DctA/DcuS interaction was shown for membrane integral wild-typic DcuS and DctA, and DcuS and a membrane integral subdomain of DctA comprising TM8 and the cytosolic helix 8b. The data implied that PAS_C_ and helix 8b of DctA play an important role in the interaction (Witan et al. 2012b). Strong interaction between PAS_C_ and helix 8b is also observed in the BACTH assay when only the corresponding cytosolic domains of DcuS and DctA are present (Fig. 6B).

If decreased interaction between transport active DctA and DcuS triggers PAS_C_ to the functional or ON state, it can be speculated that, by the same token, artificial transfer of PAS_C_ by mutation to the ON state might affect interaction between DcuS and DctA. This hypothesis was tested by the BACTH assay. Wild-typic full-length DcuS and DctA show a strong interaction in the BACTH assay that was not altered by fumarate (Fig. 6A). In contrast, for each of the two PAS_C_ type I ON mutations the β-galactosidase activity (and therefore the interaction) disappears (Fig. 6A). The response of the type II ON mutations, however, was not uniform. Whereas the interaction dropped to background levels for PAS_C_ mutations E233G and F321A (type II A), the activity was intermediate for other mutants (L228A, R322A) or even surpassed that of wild-typic DcuS (N304D) (type II B). None of all the ON mutations tested showed differences in their BACTH signal when fumarate was present. Type II B ON mutants resembled in their BACTH response wild-typic DcuS that was transferred to the ON state by fumarate and retained full interaction in this state. This again means that type I mutations infer an ON situation in DcuS with a largely changed DcuS/DctA interaction, similar to the gross reorganization of the PAS_C_ homodimer of the type I mutations. The structural basis for this ON state apparently differs from that in the physiological ON state produced by fumarate which is not decreased in DcuS/DctA interaction. In particular the type II B ON mutants appear to be more similar to the physiological (fumarate induced) ON situation of DcuS with respect to the DcuS/DctA interaction and the interaction of the PAS_C_ domains read out by the BACTH assay.

### OFF mutations in PAS_C_ with loss of DcuS function

**Figure 7:**
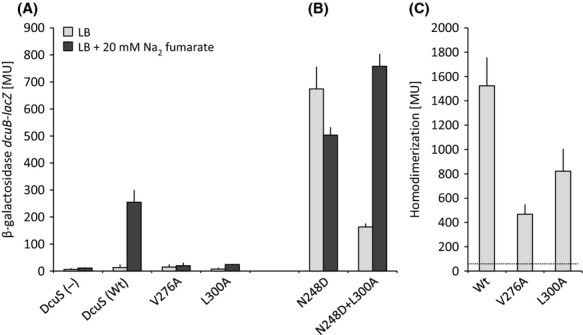
Full-length OFF variants of DcuS with mutations in the PAS_C_ domain: Effect of the OFF mutation (A) and a combined OFF/ON mutation (B) on *dcuB-lacZ* expression, and on the homodimerization (C). (A) OFF mutations in PAS_C_ (V276A and L300A variants) that have an activity of *dcuB-lacZ* expression in the noninduced (gray bar) or the induced (black bar) state below 60 Miller units. Growth was performed under anaerobic conditions in eM9 medium with glycerol plus DMSO and with or without 20 mmol/L sodium fumarate. The expression was tested in strain IMW260 (*dcuS* negative) after complementation with plasmid-encoded variants of DcuS (derivatives of pMW181). (B) The combined OFF/ON mutation with DcuS (N248D L300A) was tested as described for (A). (C) Homodimerization of full-length DcuS (wild-typic and PAS_C_ OFF mutants) was tested in the bacterial two-hybrid system (BACTH). *Escherichia coli* BTH101 was cotransformed with pairs of plasmids encoding T18-DcuS and T25-DcuS. The corresponding plasmids are derivatives of pMW429 and pMW426 (T18-DcuS and T25-DcuS, respectively) that are given in Table 1 and S1. β-galactosidase activity was measured in LB medium. The dotted line represents the background β-galactosidase activity, exceeding this threshold was considered as interaction between the corresponding T18 and T25 fusion proteins.

The sites for OFF mutations (see Fig. S4 for an overview) clustered in the N-terminal cap of PAS_C_ (five of seven OFF mutations), indicating that this region might be important in perceiving and transmitting the ON state from TM2. The OFF mutants showed a very low level of *dcuB-lacZ* expression even in the presence of fumarate (Fig. 7A), suggesting that this type of mutation maintains the inhibition of the kinase in the presence of fumarate. Interestingly, mutation of site K232 which was supposed to interact with residue Asn304 of the second monomer in homodimeric PAS_C_ and to produce an ON phenotype (Etzkorn et al. 2008) was one of the OFF mutants. OFF mutations V276A and L300A that fully inactivated full-length DcuS (Fig. 7A), inactivated also the soluble PAS_C_-kinase construct with respect to *dcuB-lacZ* expression (not shown). The proteins were produced at significant levels as concluded from interaction studies (see Fig. 7C). When PAS_C_ OFF mutations like L300A were combined with ON mutation N248D (or N304D), the double mutant showed a partial ON phenotype which was stimulated by fumarate to high levels (Fig. 7B). This indicates that the ON mutations in PAS_C_ are dominant over OFF mutations (see also Data S1 and Fig. S2).

The OFF mutants including V276A and L300A (Fig. 7C) possess substantial activity in the BACTH homodimerization assay (Fig. 7C), and a similar response was found for the corresponding PAS_C_-kinase constructs (not shown). Therefore these OFF mutants show no loss in homodimer formation.

## Discussion

### Significance of decreased PAS_C_ dimer stability for the ON phenotype

DcuS is present in the bacteria as a dimer or higher oligomer (Scheu et al. 2010b), and the dimeric state is required for autophosphorylation of the protein (Janausch et al. 2002; Scheu et al. 2010a). So far no structure of PAS_C_ from DcuS is available, but a homologous structure for the PAS1 dimer of the NifL sensor (Key et al. 2007). The previous study by Etzkorn et al. (2008) suggested that the PAS_C_ dimer functions as a hinge that transmits the signal from the transmembrane helices to the kinase. Deletion of the PAS_C_ domain renders the kinase in a constitutive active state which supports the role of PAS_C_ in signal transfer and its function as a hinge. For a more detailed analysis a series of mutations were generated in PAS_C_ that produced in contrast to the previous work three classes of ON mutations (Type I, II A, and IIB) and OFF mutations that can be differentiated when tested for function (*dcuB-lacZ* expression), dimer interaction in the BACTH assay, and interaction with the cosensor DctA. Basically, all ON mutants are active in *dcuB-lacZ* expression. Type I ON mutants show negative response in the BACTH homodimerization assay for PAS_C_ and for PAS_C_/DctA interaction. Type II A ON mutations show positive response in PAS_C_ homodimerization, and a negative response in DctA interaction. Type II B ON mutations, on the other hand are positive in the PAS_C_ homodimerization and PAS_C_/DctA interaction as well. The latter correspond in their response to the tested criteria to wild-typic DcuS after activation by fumarate.

**Figure 8:**
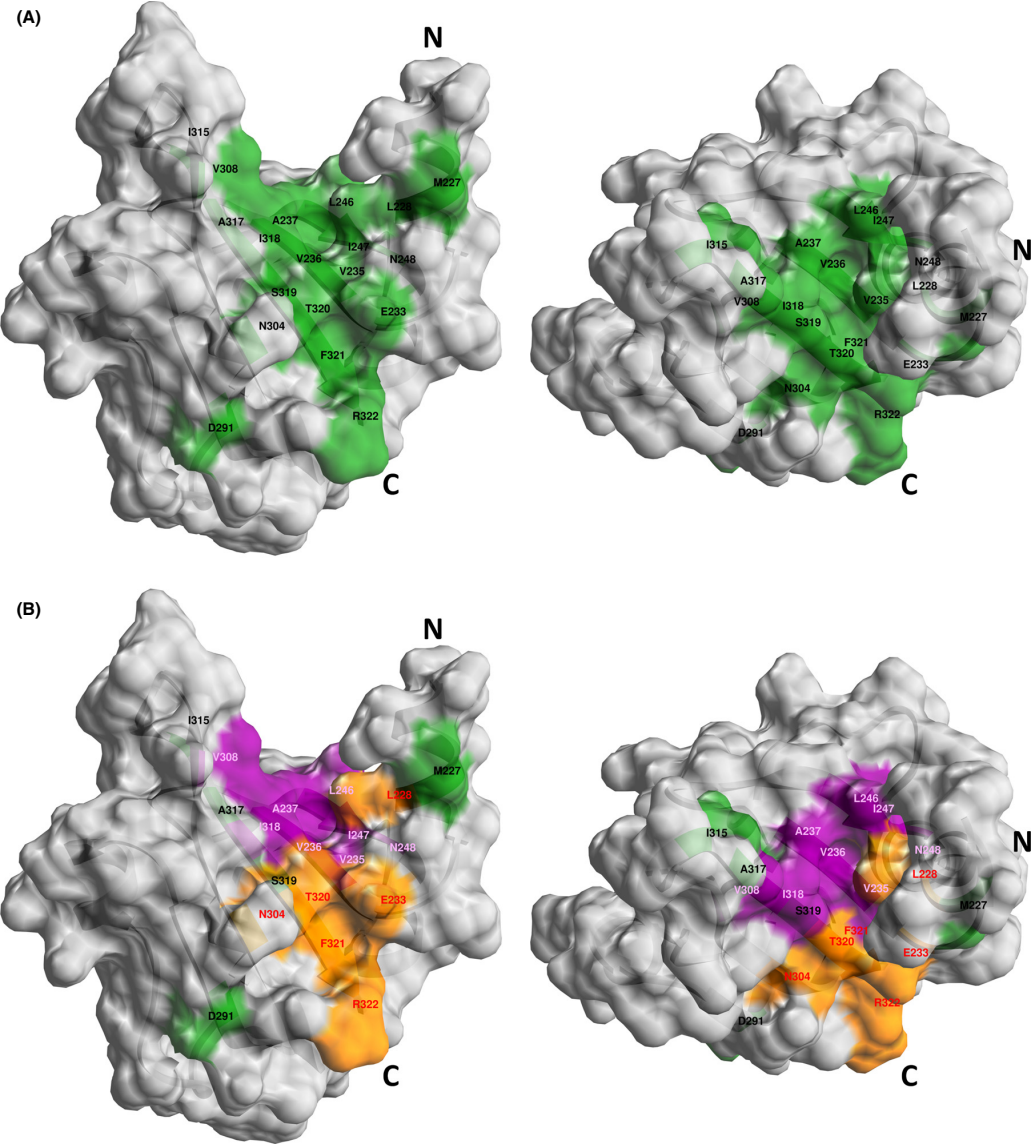
Model of the surface structure of PAS_C_ of DcuS showing the location of the ON mutations resulting in fumarate-independent expression of *dcuB-lacZ*, color coded by their influence on homodimerization. The phenotype of the mutations is shown in Figures 3A and 5. Modeling was done by the SWISS-MODEL Server using PAS1 of NifL as the template (Arnold et al. 2006). Lateral and top-down perspective of PAS_C_, the N-, and C-terminal ends are labeled. (A) All ON mutations are shown in green. (B) Purple: ON mutations that have grossly reorganized the homodimer (type I ON mutations). Orange: ON mutations that retained homodimerization (type II ON mutations). Type IIB mutations are located in the boxed area. Green: ON-mutations that were not tested for their homodimerization.

According to the data “homodimerization” of PAS_C_ is critical for the function of PAS_C_. Most of the ON mutations are located in a surface region extending from the N-terminus via a proximal cleft through to the C-terminal end of PAS_C_ (L228, E233, V235, V236, A237, L246, I247, N248, N304, V308, I318, S319, T320, F321, and R322) (Fig. 8A). The residues in and close to the cleft are predominantly hydrophobic. Only ON mutations F221I (not shown in Fig. 8A) and D291N are clearly separated from that region. Interestingly, type I and type II ON mutations cluster separately. Type I ON mutations that show grossly reorganized dimers in the BACTH assay are located at or close to the cleft (V235D, V236D, A237D, L246R, N248D, V308D, I318D (Fig. 8B, purple areas). The type II ON mutations that show no gross reorganization of the homodimer are located in the peripheral parts of the region extending to the C-terminus (L228A, E233G, N304D, T320A, F321A, and R322A) (Fig. 8B, orange areas). Type I ON mutations introduce polar or charged residues into the hydrophobic region. The negative charge apparently is not essential for the ON phenotype of residue N248 as variant N248A shows loss of interaction as well (not shown). In contrast, the ON phenotype for mutations A237D, A317D, and I318D obviously depends on the charged residue as mutations A237C, A317C/Y, and I318A are silent (Fig. S4).

**Figure 9:**
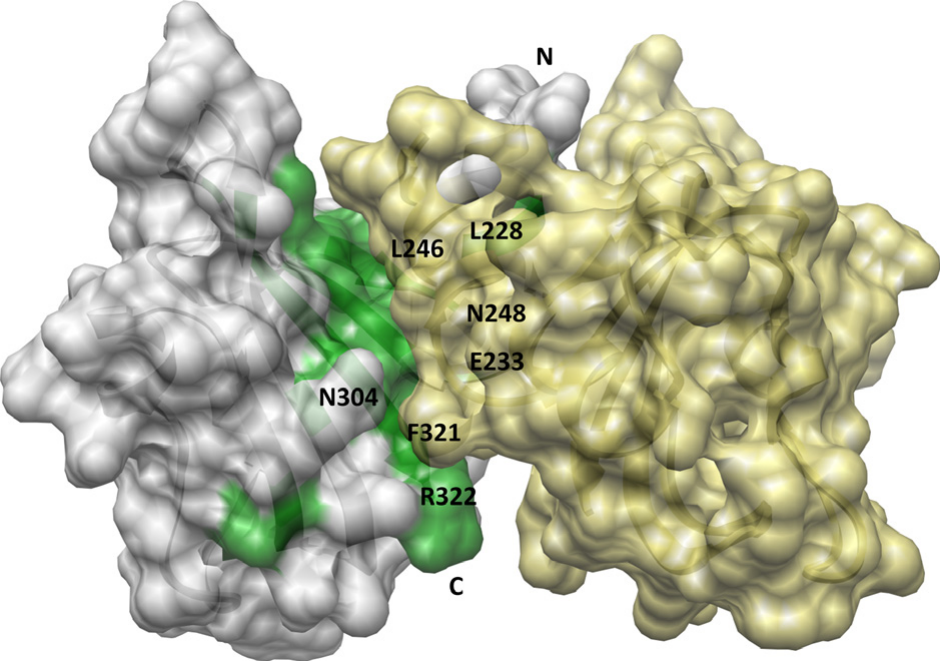
Surface structure of the DcuS PAS_C_/PAS_C_ dimer. Modeling was done by the SWISS-MODEL Server using the crystal structure of the PAS1/PAS1 dimer of NifL as the template (Arnold et al. 2006). Lateral view on the PAS_C_/PAS_C_ dimer, the N- and C-terminal ends are labeled. In monomer A (gray) residues with an ON mutation are colored in green, and for a small subset the exact position is depicted.

Similarity of PAS_C_ structure to the PAS1 dimer of NifL sensor (Key et al. 2007) suggests an interlaced structure of the PAS_C_ dimer (Fig. 9). The modeled surfaces of both PAS_C_ monomers fit closely to each other in a face to face orientation similar to the PAS1/PAS1 interaction in NifL. The contact site consists essentially of the surface region between the hydrophobic cleft and the N- and C-terminal ends of PAS_C_ (Fig. 8) and corresponds precisely to the region with the ON mutations, strongly supporting the correlation between ON-state of DcuS and PAS_C_ homodimerization state.

Residue N248 is one of the few residues strongly conserved in PAS domains (Taylor and Zhulin 1999; Etzkorn et al. 2008). The residue is in the hydrophobic surface region, but it is not exposed to the surface. For the amino and carbonyl groups of the Asn side chain, hydrogen bond contacts are predicted to backbone C- and N-atoms of V235, A251, E233, and L228 that are located in the homodimer contact region. Therefore, the role of N248 appears to be different from that of the surface residues. It could function as a hinge or relay in signal transmission to the surface of PAS_C_ and to E233 similar to the role suggested in Etzkorn et al. (2008)

### Significance of PAS_C_ for the DctA-dependent regulation of DcuS

**Figure 10:**
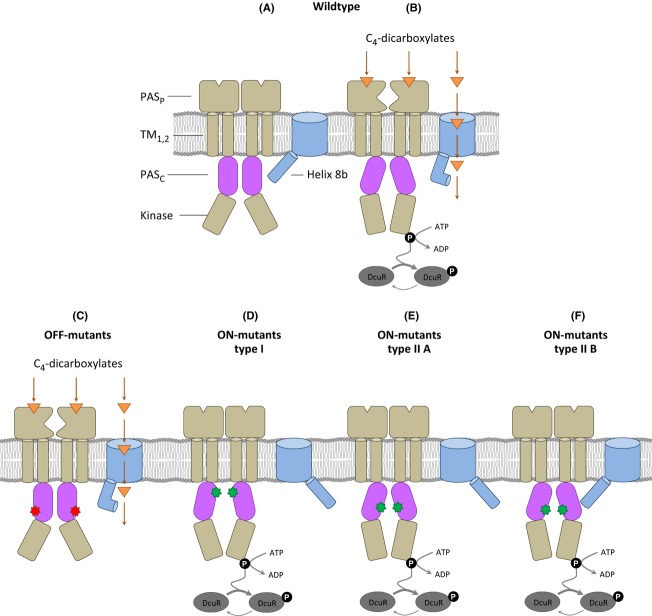
Schematic presentation of DcuS function and regulation by fumarate and the cosensor DctA. Under physiological conditions (A and B) DcuS interacts with DctA. In the absence of C_4_-dicarboxylates DctA (cytosolic helix 8b) and DcuS (PAS_C_, shown in purple) interact in a way resulting in kinase inhibition (Witan et al. 2012a,b). Through binding of C_4_-dicarboxylates (orange triangles) to DcuS and transport of them by DctA, the interaction between DctA and DcuS is relieved, resulting in loss of inhibition (B). OFF-mutations (C, red stars) within PAS_C_ prevent kinase activation irrespective of C_4_-dicarboxylate presence. In contrast, PAS_C_ ON mutations (green stars) turn on the kinase and make it C_4_-dicarboxylate independent (D–F). In PAS_C_ type I ON mutations (D) this is achieved through gross reorganization of the PAS_C_ homodimer (as described in Etzkorn et al. 2008) and resolution of the DcuS/DctA interaction. In PAS_C_ type II A ON mutations (E) the homodimerization remains unaffected but the interaction with DctA is abolished. In PAS_C_ type II B ON mutations (F) neither homodimerization nor DcuS/DctA interaction is affected as in the fumarate-induced wild type.

PAS_C_ ON mutations of type I no longer interact with DctA which is a strong functional support of the previous suggestion that PAS_C_ is important for DcuS/DctA interaction. This can be interpreted by assuming that DctA affects the functional state of DcuS by binding to PAS_C_, and vice versa, in type I ON mutations (that show also decreased DcuS/DcuS homodimerization) the structure of PAS_C_ is modified in a way that weakens or inhibits interaction with DctA. The data support the idea (Witan et al. 2012a,b) that (idle) DctA keeps PAS_C_ in the OFF state, whereas DctA engaged in transport is no longer able to keep PAS_C_ in the OFF state (Fig. 10A and B). As described earlier DcuS requires additionally direct activation by binding of fumarate to the sensor domain PAS_P_ (Zientz et al. 1998; Janausch et al. 2002; Pappalardo et al. 2003; Cheung and Hendrickson 2008; Kneuper et al. 2010; Scheu et al. 2010a). Some of the type II ON mutations, and fumarate activated DcuS, however, show no changes in DcuS/DctA interaction. It has to be explored by structural studies how big the structural changes during conversion of PAS_C_ to the physiological ON state by fumarate or by the ON mutations are. Again, the more drastic type I ON mutations are different from the physiological situation, but are very useful for studies on DctA/DcuS interaction. The presence and properties of the type I ON of DcuS which no longer interact with the inhibitor DctA strongly support the model of DctA keeping DcuS in an inactive conformation.

Thus, we come to the following propositions for the transfer of DcuS from the OFF to the ON state: No OFF state PAS_C_ mutants have been found that are affected in PAS_C_ dimerization. As OFF mutants are OFF also in the absence of DctA or DcuB (cf. Data S2 and Fig. S3), this functional state seems to implement an arrangement of the PAS_C_ dimer that does not need the stabilization by interaction with DcuB or DctA and at the same time is inflexible enough to disallow the kinases to cross-phosphorylate each other.

For mutants that cause the ON state we have three classes: In type I ON mutants, the PAS_C_ dimer interface is grossly reorganized and the interaction with DctA is weakened as well. In type II ON mutants the PAS_C_ interface is only mildly changed if at all as in the activated wild type. Two of these mutants (E233G and F321A, type IIA) have a strongly reduced ability to bind DctA thus it cannot be excluded that the ON state is induced by the impossibility for DctA to inactivate these mutants. The other subclass of type II mutants (L228A, N304D, R322A, type IIB) retain the interface and the interaction with DctA. These mutations have the most similar behavior to fumarate activated wild-typic DcuS, which in this nomenclature would be most similar to the type IIB ON mutants L228A, N304D, and R322A. Therefore, these three mutants are interesting candidates for further in-depth investigations in the search for the mechanism of activation of DcuS.

### A model for the role of PAS_C_ in DcuS function

Regarding PAS_C_ and the control of its functional state five different situations are shown in Figure 10. (1) The wild type in the OFF state is stabilized by DctA and the absence of effector (fumarate). (2) Addition of effector or removal of DctA activates the kinase, and the His residues become phosphorylated (ON state). The PAS_P_ domains change their structures which was shown by X-ray on DcuS and CitA, and also change the state of the transmembrane helix TM2. The question how the PAS_C_ domains contribute to the ON/OFF switch was the topic of the mutation study. The ON state could either make the kinases more flexible, for example, by grossly reorganizing the PAS_C_ interactions or drive the conformation of the PAS_C_ dimer into a conformation which arranges the kinases such that they can trans-phosphorylate (mild change). In contrast, OFF mutants stay in the OFF state despite the activation by fumarate. They all exhibit PAS_C_ in a dimeric state which is required for silencing the kinase. The PAS_C_ OFF conformation adopted in the mutant is assumed to be similar to the wild-type form induced by DctA and the absence of effector.

ON mutants can be implemented in three different ways: Type I ON mutants grossly reorganize in the PAS_C_ dimer and might even show a localized disruption of the PAS_C_ dimer in the full-length construct and in the truncated PAS_C_-kinase construct. It is reasonable to assume that the reorganized dimer allows more flexibility for the kinases and release of silencing by PAS_C_. Type II A ON mutants maintain the PAS_C_ dimer interaction, but do not interact with DctA (like the type I ON mutations). This is the same situation as in bacteria with wild-typic DcuS but lacking DctA in deletion strains. This is sufficient for activation even in the absence of fumarate. Whether the type II A ON mutants only affect the interaction to DctA or also mildly reorganize the PAS_C_ dimer is unresolved. The type II B ON mutants show the native interaction with DctA but do not need activation by fumarate. They are still dimers in full-length DcuS as well as in the PAS_C_-kinase construct. Most probably, the type II B ON mutations resemble in the functional ON state, PAS_C_ dimer conformation and PAS_C_/DctA dimer interaction the situation present during fumarate activation of wild-typic DcuS. Structure investigations of these mutants might therefore pave the way to study the structure of the PAS_C_ domain in the activated state.

## Supporting Information

**Figure S1.** Western-blots of T18- and T25-fusion proteins. Proteins were expressed under conditions comparable to the respective experiment and subjected to SDS-PAGE and Western-blotting. (A) Immunostained with antiserum against DcuS-PAS_P_. 1: JM109; 2: DcuS; 3: T18-DcuS(N248D); 4: T18-DcuS(L246R); 5: T18-DcuS(V235D); 6: T18-DcuS(V236D); 7: T18-DcuS(A23 7D); 8: T18-DcuS(I318D). (B) Immunostained with antiserum against DcuS-PAS_P_. 1: JM109; 2: DcuS; 3: T25-DcuS(N248D); 4: T25-DcuS(L246R); 5: T25-DcuS(V235D); 6: T25-DcuS(V236D); 7: T25-DcuS(A237D); 8: T25-DcuS(I318D). (C) Immunostained with anti-His antibody. 1: JM109; 2: His6-PAS_C_-T18 (pMW1657); 4: His6-Kinase-T18 (pMW1659). (D) Immunostained with anti-His antibody. 1: JM109; 2: His6-PAS_C_-T25 (pMW1656); 4: His6-Kinase-T25 (pMW1658).

**Figure S2.** Expression of *dcuB-lacZ* in a DcuS OFF (H110A, or L300A) and a silent mutant (Q229A) in wild-type background and in combination with ON mutation N248D. Expression is compared to wild-typic and ON-variant N248D of DcuS. Gray and black bars show the induction after anaerobic growth in eM9 with glycerol plus DMSO, without or with sodium fumarate (20 mmol/L), respectively. The expression was tested in strain IMW260 (*dcuS* negative) after complementation with plasmid encoded DcuS and variants (plasmid pMW181 and derivatives, see Tables 1 and S1).

**Figure S3.** Expression of *dcuB-lacZ* in DcuS ON (N248D) or OFF (H110A, or V276A) variants in *dcuB* mutant background. Expression of *dcuB-lacZ* was tested in strain IMW536 (*dcuB* and *dcuS* negative) after complementation with plasmid encoded variants of DcuS (N248D, V276A, or H110A) and of DcuB (Wt [wild-type] DcuB negative and D398N variant). Gray and black bars show the induction after anaerobic growth in eM9 with glycerol plus DMSO, without or with sodium fumarate (20 mmol/L), respectively. The plasmid encoded DcuS and DcuB variants were encoded by plasmids pMW181 and pMW228 and derivatives, respectively (see Tables 1 and S1).

**Figure S4.** Overview of ON (green arrows), OFF (red arrows) and silent (grey arrows) mutations in the PAS_C_ domain. The figure gives a summary of all ON and OFF mutations with the location and type of mutation in PAS_C_. For the definition of ON and OFF mutations see Figures 3 and 8, respectively. See Tables 1 and S1 for the corresponding plasmids.

**Data S1.** ON mutations in PASC have a dominant effect on kinase activity.

**Data S2.** PAS_C_ and its role in co-sensing of DcuB and DctA.

**Table S1.** Variants of *Escherichia coli* plasmids, listed in Table 1.
