# Supplementary figures and images for: The cytoplasmic PASC domain of the sensor kinase DcuS of Escherichia coli: role in signal transduction, dimer formation, and DctA interaction
Source: Microbiologyopen. 2013 Sep 9;2(6):912–27. doi: 10.1002/mbo3.127 (PMC3892338; doi:10.1002/mbo3.127)

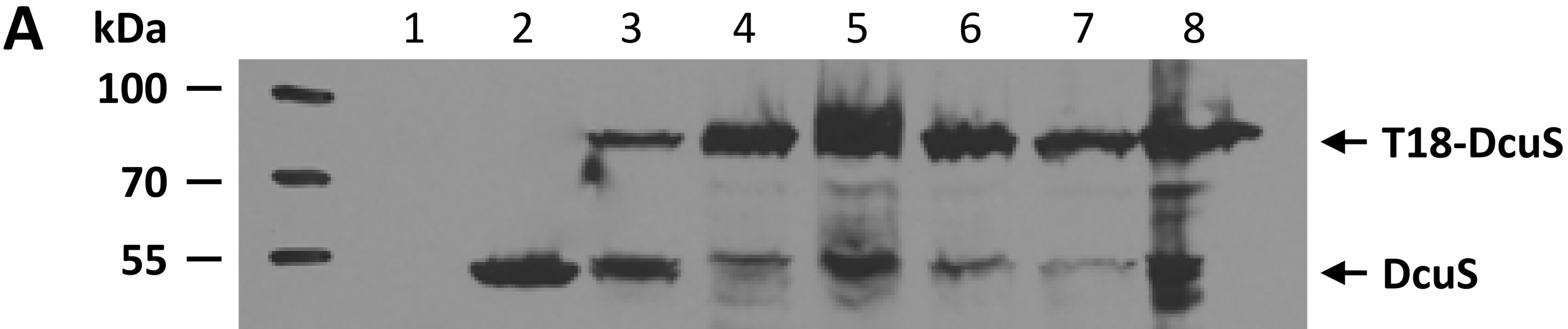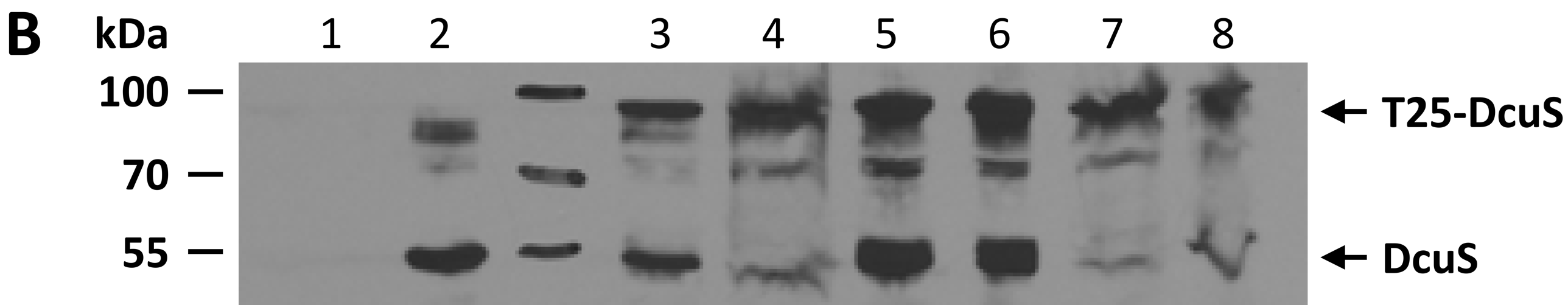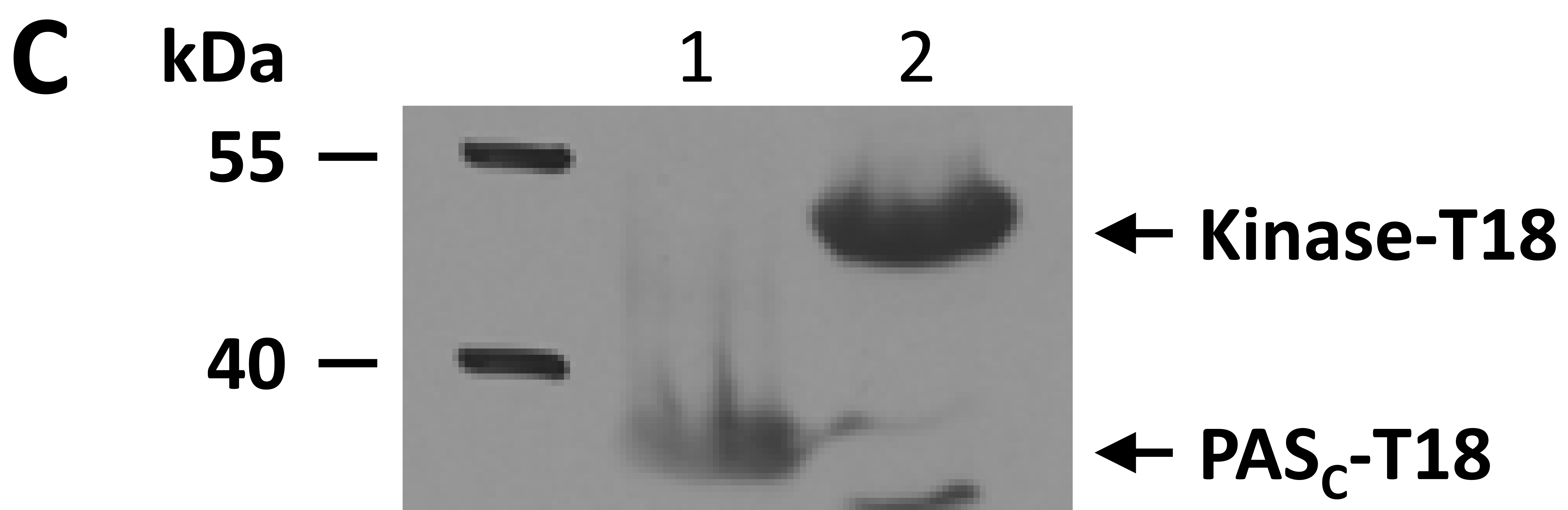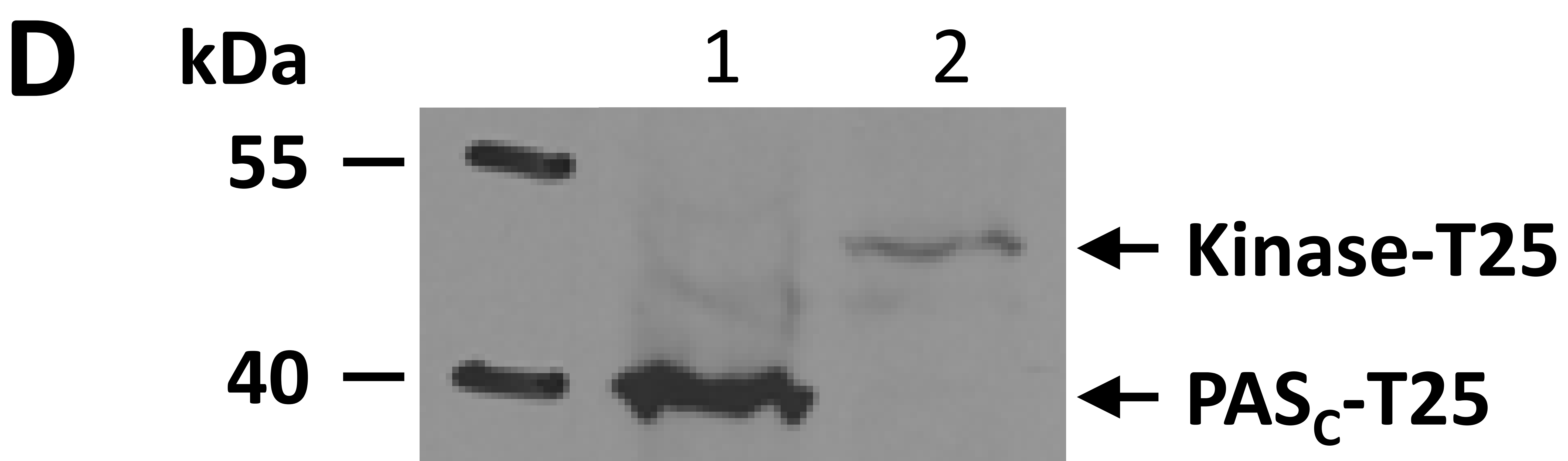

Supplement: Supplementary file 1 [file mbo30002-0912-SD1.pdf]

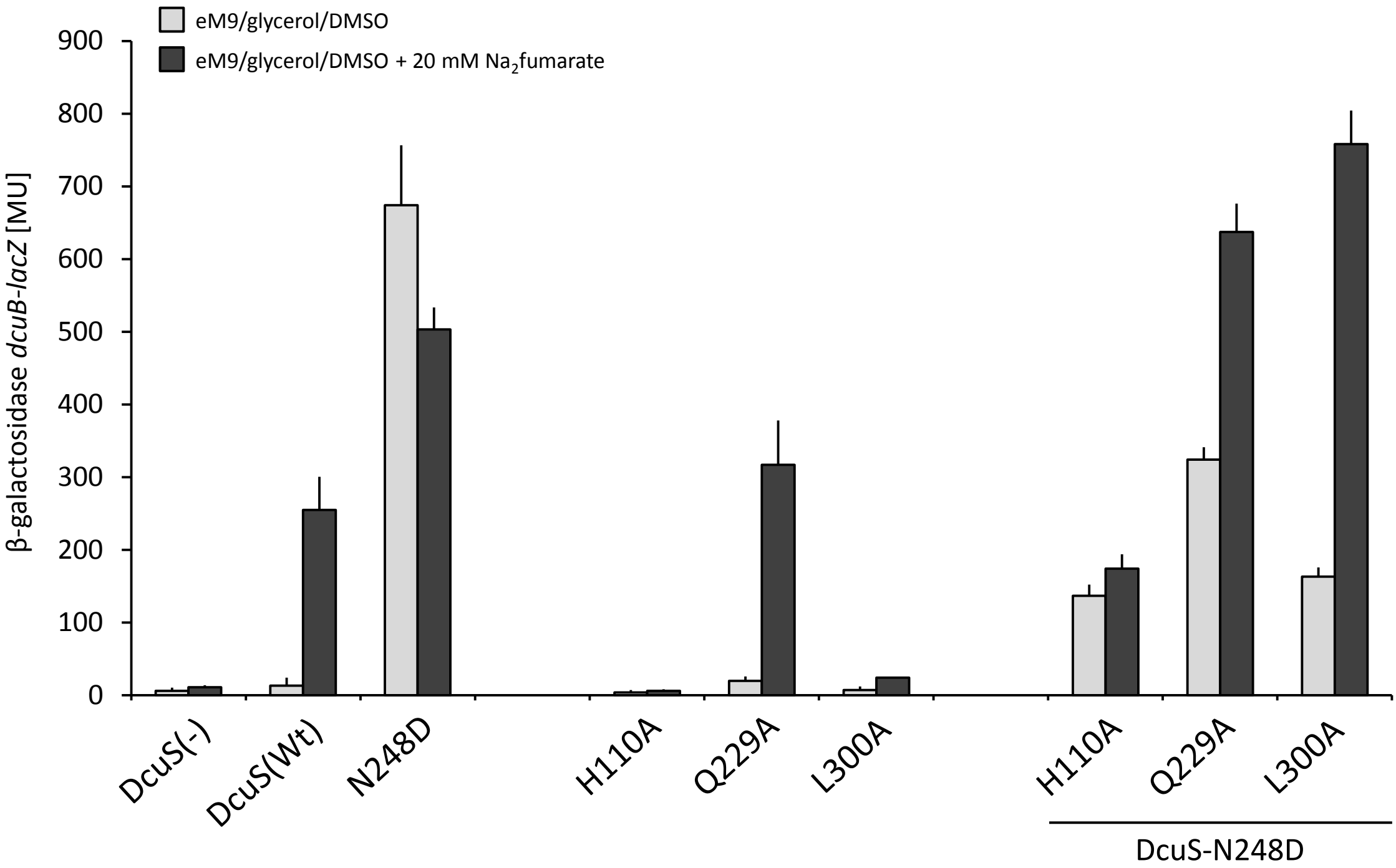

Supplement: Supplementary file 2 [file mbo30002-0912-SD2.pdf]

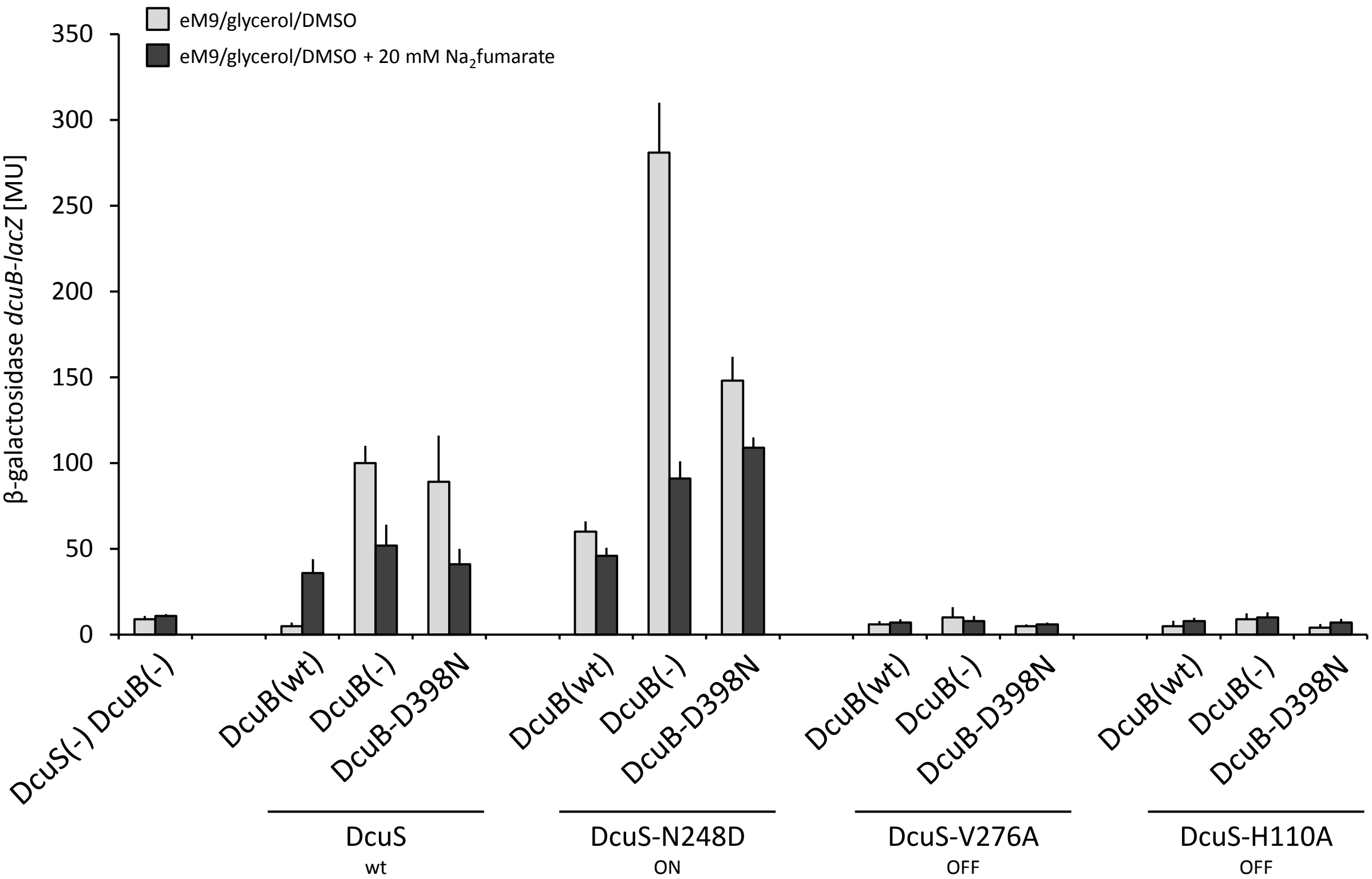

Supplement: Supplementary file 3 [file mbo30002-0912-SD3.pdf]

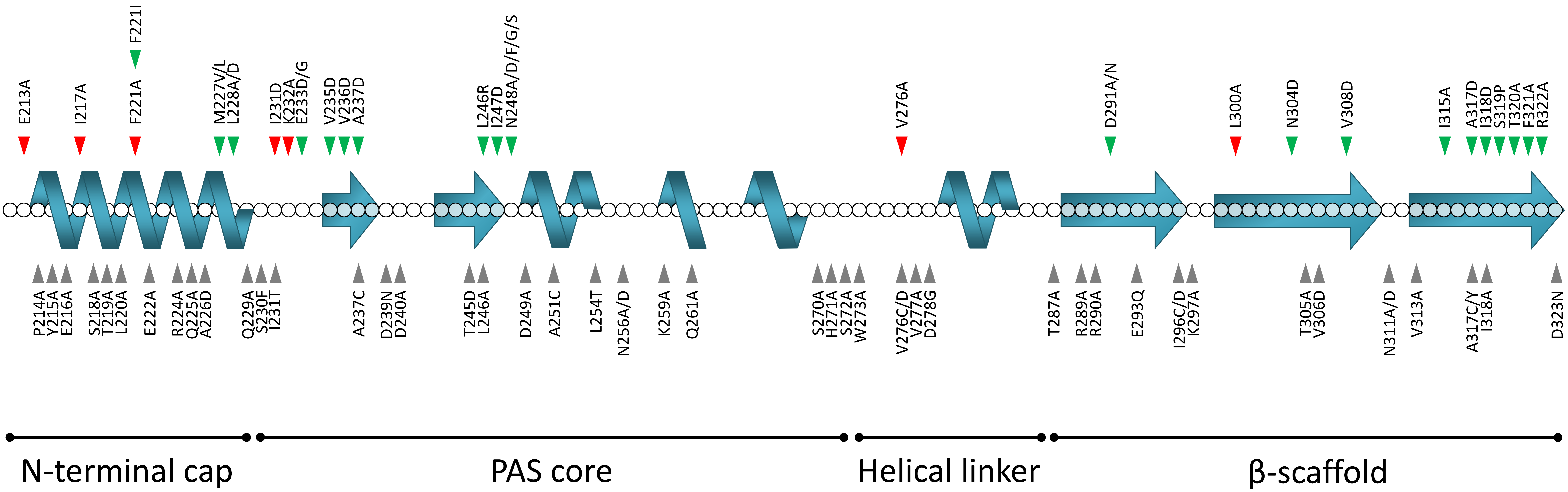

Supplement: Supplementary file 4 [file mbo30002-0912-SD4.pdf]
