# Supplementary material for: The cytoplasmic PASC domain of the sensor kinase DcuS of Escherichia coli: role in signal transduction, dimer formation, and DctA interaction
Source: Microbiologyopen. 2013 Sep 9;2(6):912–27. doi: 10.1002/mbo3.127 (PMC3892338; doi:10.1002/mbo3.127)
Supplement: Supplementary file 5 [file mbo30002-0912-SD5.doc]

**The cytoplasmic PAS_C_ domain of the sensor kinase DcuS of *Escherichia coli*: Role in signal transduction, dimer formation and DctA interaction**

Christian Monzel,*^1^ Pia Degreif-Dünnwald,*^1^ Christina Gröpper,^1^ Christian Griesinger,^2^ Gottfried Unden^1^

**Supplemental information**

**ON mutations in PAS_C_ have a dominant effect on kinase activity**

ON and OFF mutations in the PAS_P_ and PAS_C_ domains of DcuS were introduced by directed mutation into the same DcuS protein and then tested for their effect on activity. Thus various mutations were introduced into the (constitutive) PAS_C_ ON mutant N248D, and the double mutants were assayed for their activity in *dcuB-lacZ* expression (Fig. S2). Mutation DcuS(H110A) is defect in the C_4_-dicarboxylate binding site of PAS_P_ and completely devoid of fumarate stimulated *dcuB-lacZ* expression (Kneuper *et al.*, 2005). Combination of H110A OFF mutation with the N248D ON mutation restored expression of *dcuB-lacZ* expression that was slightly stimulated by fumarate. The stimulated activity corresponded to 68% of wild-typic DcuS. In the same way, combination of mutation H110A with ON mutation N304D resulted in a variant (H110A N304D) with wild-typic activities (105%) in the fumarate induced state DcuS (not shown). Combination of mutation N248D with OFF mutation L300A (located in PAS_C_), yielded a variant with high activity and a (partial) ON phenotype. Combinations of mutation N248D with silent mutations like Q229A retained the ON phenotype of N248D. Overall, it appears that ON mutations in PAS_C_ are dominant over OFF mutations in PAS_P_ or PAS_C_. Double mutants carrying OFF mutation H110A plus the silent mutation L220A, or H110A plus the OFF mutations V276A or L300A were completely inactive in expressing *dcuB-lacZ*, as expected (not shown).

**PAS_C_ and its role in co-sensing of DcuB and DctA**

The C_4_-dicarboxylate transporter DctA and the fumarate/succinate antiporter DcuB function as co-sensors of DcuS and are required for wild-typic response of DcuS to C_4_-dicarboxylates (Witan *et al*., 2012a; Kleefeld *et al.*, 2009; Davies *et al.*, 1999). It was tested whether DcuS-PAS_C_ ON or OFF variants are affected in their response of *dcuB-lacZ* expression to the availability of DcuB. Strain IMW536 that is deficient for DcuS and DcuB was transformed with plasmids encoding variants of DcuS and DcuB (Fig. S3). When only DcuS was present, the expression of *dcuB-lacZ* was maximal without fumarate as described earlier (Kleefeld *et al.*, 2009), and fumarate had even an inhibitory effect. By introducing plasmid-encoded DcuB the expression became fumarate dependent again, whereas introducing the regulatory deficient forms of DcuB (mutations in D398N, K353A or T394I) restored the capability for growth, but not for repression of *dcuB-lacZ* in the absence of fumarate (Fig. S3). When the complementation was performed with the DcuS ON variant DcuS(N248D), expression of *dcuB-lacZ* was constitutive in the absence and presence of DcuB. The response was basically the same when the regulatory mutants DcuB D398N (Fig. S3) or T394A and K353A replaced wild-typic DcuB (not shown). The activity was highest, however, in the combination of the DcuS ON mutant and DcuB deficiency, indicating some additive effect of the ON mutation and DcuB deficiency. When the same experiments were performed with the DcuS ON variant DcuS(N304D), similar results as with DcuS(N248D) were obtained (not shown). Overall, this means that DcuS(N248D) (and DcuS(N304D)) are always in the ON state, irrespective of the presence of DcuB and of the DcuB phenotype. DcuB therefore has no longer a regulatory role on DcuS.

The DcuS OFF mutant DcuS(V276) which is not stimulated by fumarate (see Fig. 4), did not regain activity in *dcuB-lacZ* expression when DcuB was deleted, or when regulatory incompetent mutants of DcuB were present (Fig. S3). In the DcuS(H110A) mutant which is in the OFF state as well, neither deletion of DcuB nor conversion of DcuB to regulatory incompetent forms (e.g. DcuB(N398N)) was able to respond to fumarate (Fig. S3), indicating that neither the DcuS(V276A) nor the DcuS(H110A) variant were able to respond to DcuB.

**Table S1**. Variants of *E. coli* plasmids, listed in Table 1.

| Plasmid | Genotype | Reference or source |
| --- | --- | --- |
| Plasmids for reporter gene measurements | | |
| pMW236 | pMW181 but DcuS-H110A (Kan^r^) | (Krämer *et al.*, 2007) |
| pMW405 | pMW228 but DcuB-D398N | (Kleefeld *et al*. 2009) |
| pMW420 | pMW181 but DcuS-D239N (Kan^r^) | This study |
| pMW421 | pMW181 but DcuS-N248D (Kan^r^) | This study |
| pMW422 | pMW181 but DcuS-K259A (Kan^r^) | This study |
| pMW474 | pMW181 but DcuS-A251C (Kan^r^) | This study |
| pMW475 | pMW181 but DcuS-T287A (Kan^r^) | This study |
| pMW476 | pMW181 but DcuS-R290A (Kan^r^) | This study |
| pMW477 | pMW181 but DcuS-D291N (Kan^r^) | This study |
| pMW478 | pMW181 but DcuS-K297A (Kan^r^) | This study |
| pMW479 | pMW181 but DcuS-T305A (Kan^r^) | This study |
| pMW480 | pMW181 but DcuS-S270A (Kan^r^) | This study |
| pMW481 | pMW181 but DcuS-W273A (Kan^r^) | This study |
| pMW482 | pMW181 but DcuS-Q261A (Kan^r^) | This study |
| pMW483 | pMW181 but DcuS-N248S (Kan^r^) | This study |
| pMW484 | pMW181 but DcuS-N248G (Kan^r^) | This study |
| pMW485 | pMW181 but DcuS-N248A (Kan^r^) | This study |
| pMW486 | pMW181 but DcuS-N248F (Kan^r^) | This study |
| pMW487 | pMW181 but DcuS-D291A (Kan^r^) | This study |
| pMW488 | pMW181 but DcuS-N304D (Kan^r^) | This study |
| pMW489 | pMW181 but DcuS-L254T (Kan^r^) | This study |
| pMW490 | pMW181 but DcuS-R289A (Kan^r^) | This study |
| pMW491 | pMW181 but DcuS-E293Q (Kan^r^) | This study |
| pMW558 | pMW181 but DcuS-H110A-N248D (Kan^r^) | This study |
| pMW631 | pMW181 but DcuS-Y215A (Kan^r^) | This study |
| pMW632 | pMW181 but DcuS-Q229A (Kan^r^) | This study |
| pMW633 | pMW181 but DcuS-K232A (Kan^r^) | This study |
| pMW634 | pMW181 but DcuS-D240A (Kan^r^) | This study |
| pMW636 | pMW181 but DcuS-A317C (Kan^r^) | This study |
| pMW637 | pMW181 but DcuS-A317Y (Kan^r^) | This study |
| pMW638 | pMW181 but DcuS-A317D (Kan^r^) | This study |
| pMW640 | pMW181 but DcuS-I296C (Kan^r^) | This study |
| pMW641 | pMW181 but DcuS-I296D (Kan^r^) | This study |
| pMW667 | pMW181 but DcuS-V276A (Kan^r^) | This study |
| pMW668 | pMW181 but DcuS-V276C (Kan^r^) | This study |
| pMW669 | pMW181 but DcuS-V276D (Kan^r^) | This study |
| pMW712 | pMW181 but DcuS-N311A (Kan^r^) | This study |
| pMW713 | pMW181 but DcuS-N311D (Kan^r^) | This study |
| pMW734 | pMW181 but DcuS-Q229A-N248D (Kan^r^) | This study |
| pMW748 | pMW181 but DcuS-D323N (Kan^r^) | This study |
| pMW810 | pMW181 but DcuS-N256A (Kan^r^) | This study |
| pMW811 | pMW181 but DcuS-N256D (Kan^r^) | This study |
| pMW812 | pMW181 but DcuS-H271A (Kan^r^) | This study |
| pMW815 | pMW181 but DcuS-V277A (Kan^r^) | This study |
| pMW868 | pMW181 but DcuS-I231T (Kan^r^) | This study |
| pMW913 | pMW181 but DcuS-L228A (Kan^r^) | This study |
| pMW914 | pMW181 but DcuS-I318A (Kan^r^) | This study |
| pMW920 | pMW181 but DcuS-L246R (Kan^r^) | This study |
| pMW921 | pMW181 but DcuS-L246A (Kan^r^) | This study |
| pMW922 | pMW181 but DcuS-L300A (Kan^r^) | This study |
| pMW923 | pMW181 but DcuS-V313A (Kan^r^) | This study |
| pMW924 | pMW181 but DcuS-A237C (Kan^r^) | This study |
| pMW925 | pMW181 but DcuS-S319P (Kan^r^) | This study |
| pMW936 | pMW181 but DcuS-E213A (Kan^r^) | This study |
| pMW937 | pMW181 but DcuS-P214A (Kan^r^) | This study |
| pMW938 | pMW181 but DcuS-E216A (Kan^r^) | This study |
| pMW939 | pMW181 but DcuS-I217A (Kan^r^) | This study |
| pMW940 | pMW181 but DcuS-L220A (Kan^r^) | This study |
| pMW941 | pMW181 but DcuS-F221A (Kan^r^) | This study |
| pMW942 | pMW181 but DcuS-E222A (Kan^r^) | This study |
| pMW943 | pMW181 but DcuS-Q225A (Kan^r^) | This study |
| pMW944 | pMW181 but DcuS-D249A (Kan^r^) | This study |
| pMW945 | pMW181 but DcuS-T320A (Kan^r^) | This study |
| pMW946 | pMW181 but DcuS-F321A (Kan^r^) | This study |
| pMW947 | pMW181 but DcuS-R322A (Kan^r^) | This study |
| pMW956 | pMW181 but DcuS-S218A (Kan^r^) | This study |
| pMW957 | pMW181 but DcuS-T219A (Kan^r^) | This study |
| pMW958 | pMW181 but DcuS-S272A (Kan^r^) | This study |
| pMW959 | pMW181 but DcuS-I315A (Kan^r^) | This study |
| pMW965 | pMW181 but DcuS-D278G (Kan^r^) | This study |
| pMW1168 | pMW181 but Δ209-324 (DcuS-ΔPASc) | This study |
| pMW1169 | pMW181 but Δ209-233 (EX1-2) | This study |
| pMW1170 | pMW181 but Δ209-253 (EX1-3) | This study |
| pMW1171 | pMW181 but Δ209-285 (EX1-4) | This study |
| pMW1172 | pMW181 but Δ285-324 (EX4-5) | This study |
| pMW1173 | pMW181 but Δ253-324 (EX3-5) | This study |
| pMW1174 | pMW181 but Δ233-324 (EX2-5) | This study |
| pMW1386 | pMW181 but DcuS-N248D-L300A (Kan^r^) | This study |
| pMW1389 | pMW181 but DcuS-R224A (Kan^r^) | This study |
| pMW1508 | pMW181 but DcuS-S230F (Kan^r^) | This study |
| pMW1555 | pMW181 but DcuS-E233G (Kan^r^) | This study |
| pMW1556 | pMW181 but DcuS-V235D (Kan^r^) | This study |
| pMW1557 | pMW181 but DcuS-V236D (Kan^r^) | This study |
| pMW1720 | pMW181 but DcuS-F221I (Kan^r^) | This study |
| pMW1725 | pMW181 but DcuS-M227L (Kan^r^) | This study |
| pMW1727 | pMW181 but DcuS-M227V (Kan^r^) | This study |
| pMW1729 | pMW181 but DcuS-E233D (Kan^r^) | This study |
| pMW1757 | pMW181 but DcuS-A237D (Kan^r^) | This study |
| pMW1758 | pMW181 but DcuS-T245D (Kan^r^) | This study |
| pMW1759 | pMW181 but DcuS-I318D (Kan^r^) | This study |
| pMW1813 | pMW181 but DcuS-A226D (Kan^r^) | This study |
| pMW1816 | pMW181 but DcuS-L228D (Kan^r^) | This study |
| pMW1819 | pMW181 but DcuS-I231D (Kan^r^) | This study |
| pMW1822 | pMW181 but DcuS-I247D (Kan^r^) | This study |
| pMW1825 | pMW181 but DcuS-V306D (Kan^r^) | This study |
| pMW1828 | pMW181 but DcuS-V308D (Kan^r^) | This study |
|  | | |
| Plasmids for BACTH measurements | | |
| pMW1011 | pMW426 but DcuS-N248D (Kan^r^) | This study |
| pMW1012 | pMW429 but DcuS-N248D (Amp^r^) | This study |
| pMW1013 | pMW426 but DcuS-N304D (Kan^r^) | This study |
| pMW1014 | pMW429 but DcuS-N304D (Amp^r^) | This study |
| pMW1015 | pMW426 but DcuS-V276A (Kan^r^) | This study |
| pMW1016 | pMW429 but DcuS-V276A (Amp^r^) | This study |
| pMW1019 | pMW426 but DcuS-L300A (Kan^r^) | This study |
| pMW1020 | pMW429 but DcuS-L300A (Amp^r^) | This study |
| pMW1029 | pMW429 but DcuS-L228A (Amp^r^) | This study |
| pMW1030 | pMW426 but DcuS-L228A (Kan^r^) | This study |
| pMW1031 | pMW429 but DcuS-R322A (Amp^r^) | This study |
| pMW1032 | pMW426 but DcuS-R322A (Kan^r^) | This study |
| pMW1564 | pMW429 but DcuS-L246R (Amp^r^) | This study |
| pMW1565 | pMW426 but DcuS-L246R (Kan^r^) | This study |
| pMW1569 | pMW1075 but DcuS-L228A (Kan^r^) | This study |
| pMW1571 | pMW1075 but DcuS-L246R (Kan^r^) | This study |
| pMW1573 | pMW1075 but DcuS-N248D (Kan^r^) | This study |
| pMW1575 | pMW1075 but DcuS-L300A (Kan^r^) | This study |
| pMW1577 | pMW1075 but DcuS-N304D (Kan^r^) | This study |
| pMW1618 | pMW429 but DcuS-V235D (Amp^r^) | This study |
| pMW1619 | pMW426 but DcuS-V235D (Kan^r^) | This study |
| pMW1620 | pMW429 but DcuS-V236D (Amp^r^) | This study |
| pMW1621 | pMW426 but DcuS-V236D (Kan^r^) | This study |
| pMW1633 | pMW1075 but DcuS-V235D (Kan^r^) | This study |
| pMW1637 | pMW1075 but DcuS-R322A (Kan^r^) | This study |
| pMW1639 | pMW1075 but DcuS-V276A (Kan^r^) | This study |
| pMW1686 | pMW429 but DcuS-E233G (Amp^r^) | This study |
| pMW1687 | pMW426 but DcuS-E233G (Kan^r^) | This study |
| pMW1735 | pMW429 but DcuS-T320A (Amp^r^) | This study |
| pMW1736 | pMW426 but DcuS-T320A (Kan^r^) | This study |
| pMW1737 | pMW429 but DcuS-F321A (Amp^r^) | This study |
| pMW1738 | pMW426 but DcuS-F321A (Kan^r^) | This study |
| pMW1760 | pMW429 but DcuS-A237D (Amp^r^) | This study |
| pMW1761 | pMW426 but DcuS-A237D (Kan^r^) | This study |
| pMW1764 | pMW429 but DcuS-I318D (Amp^r^) | This study |
| pMW1765 | pMW426 but DcuS-I318D (Kan^r^) | This study |
| pMW1823 | pMW429 but DcuS-I247D (Amp^r^) | This study |
| pMW1824 | pMW426 but DcuS-I247D (Kan^r^) | This study |
| pMW1829 | pMW429 but DcuS-V308D (Amp^r^) | This study |
| pMW1830 | pMW426 but DcuS-V308D (Kan^r^) | This study |

**Fig. S1**. Westernblots mit anti-PAS_P_


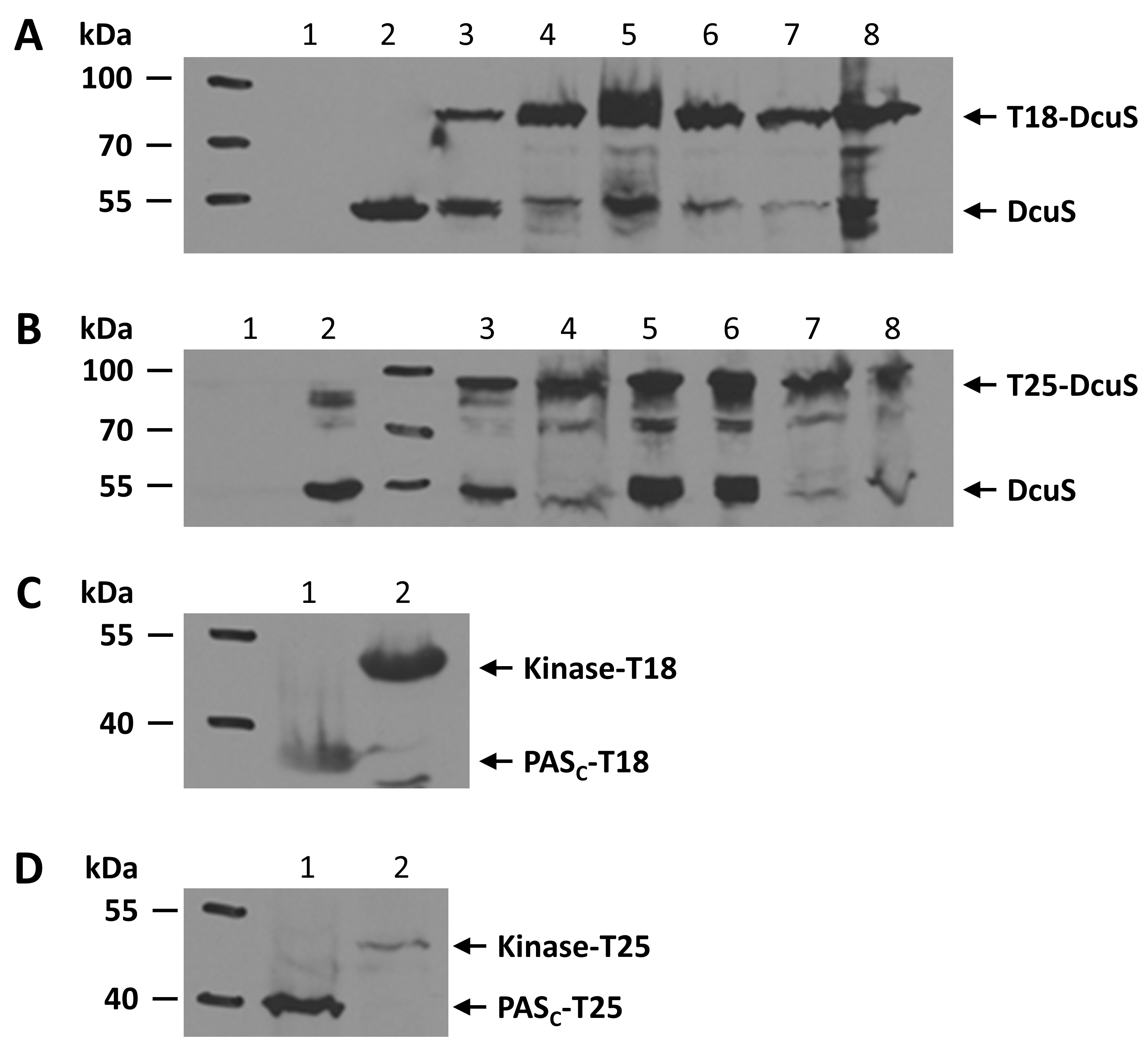


**Figure S1. Western-Blot of T18- and T25-fusion proteins.** Proteins were expressed under conditions comparable to the respective experiment and subjected to SDS-PAGE and Western Blot. (A) Immunostained with Antiserum against DcuS-PAS_P_. 1: JM109. 2: DcuS. 3: T18-DcuS(N248D). 4: T18-DcuS(L246R). 5: T18-DcuS(V235D). 6: T18-DcuS(V236D). 7: T18-DcuS(A237D). 8: T18-DcuS(I318D). (B) Immunostained with Antiserum against DcuS-PAS_P_. 1: JM109. 2: DcuS. 3: T25-DcuS(N248D). 4: T25-DcuS(L246R). 5: T25-DcuS(V235D). 6: T25-DcuS(V236D). 7: T25-DcuS(A237D). 8: T25-DcuS(I318D). (C) Immunostained with Anti-His antibody. 1: JM109. 2: His6-PAS_C_-T18 (pMW1657). 4: His6-Kinase-T18 (pMW1659). (D) Immunostained with Anti-His antibody. 1: JM109. 2: His6-PAS_C_-T25 (pMW1656). 4: His6-Kinase-T25 (pMW1658).

**Figure S2**

**
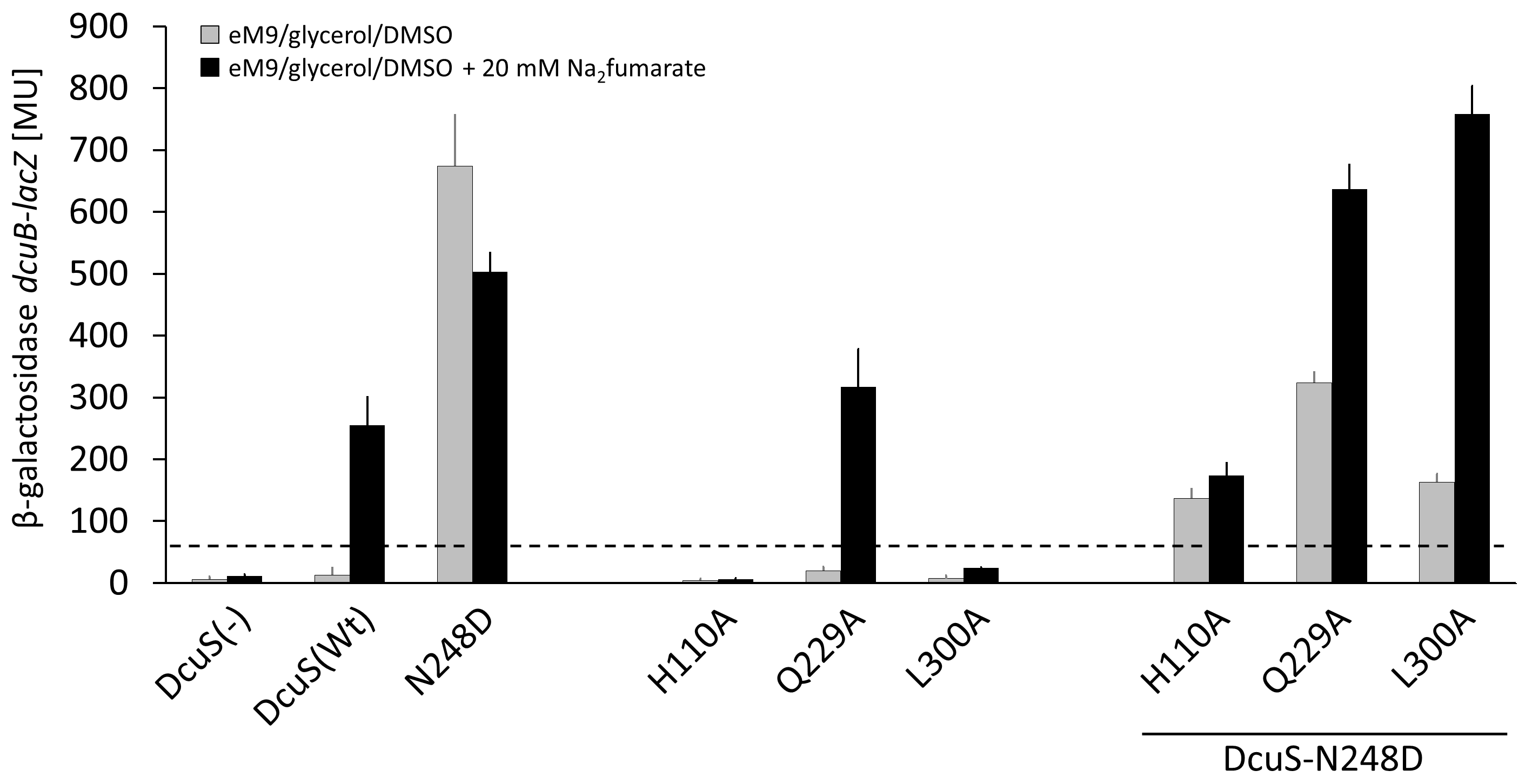
**

**Figure S2. Expression of *dcuB-lacZ* in a DcuS OFF (H110A, or L300A) and a silent mutant (Q229A) in wild-typ background and in combination with ON mutation N248D.** Expression is compared to wild-typic and ON-variant N248D of DcuS. The brocken line gives the limit for ON mutations (see Fig. 3). Grey and black bars show the induction after anaerobic growth in eM9 with glycerol plus DMSO, without or with sodium fumarate (20 mM), respectively. The expression was tested in strain IMW260 (*dcuS* negative) after complementation with plasmid encoded DcuS and variants (plasmid pMW181 and derivatives, see Table 1).

**Figure S3**


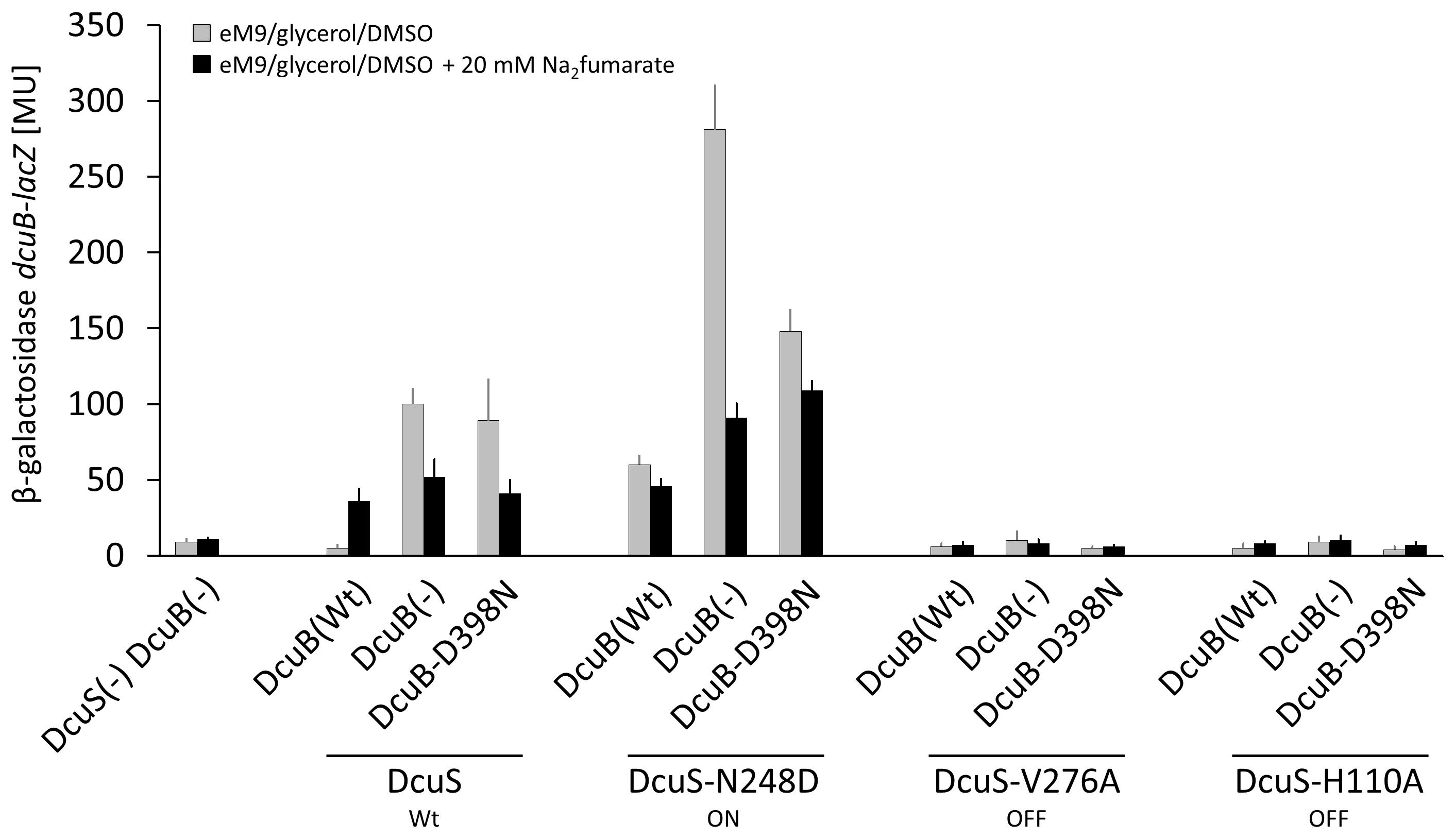


**Figure S3. Expression of *dcuB-lacZ* in DcuS ON (N248D) or OFF (H110A, or V276A) variants in *dcuB* mutant background.** Expression of *dcuB-lacZ* was tested strain IMW536 (*dcuB* and *dcuS* negative) after complementation with plasmid encoded variants of DcuS (N248D, V276A, or H110A) and of DcuB (Wt (wild-type) DcuB negative and D398N variant). Grey and black bars show the induction after anaerobic growth in eM9 with glycerol plus DMSO, without or with sodium fumarate (20 mM), respectively. The plasmid encoded DcuS and DcuB variants were encoded by plasmids pMW181 and pMW228 and derivatives, respectively (see Table 1).

**Figure S4.**

**
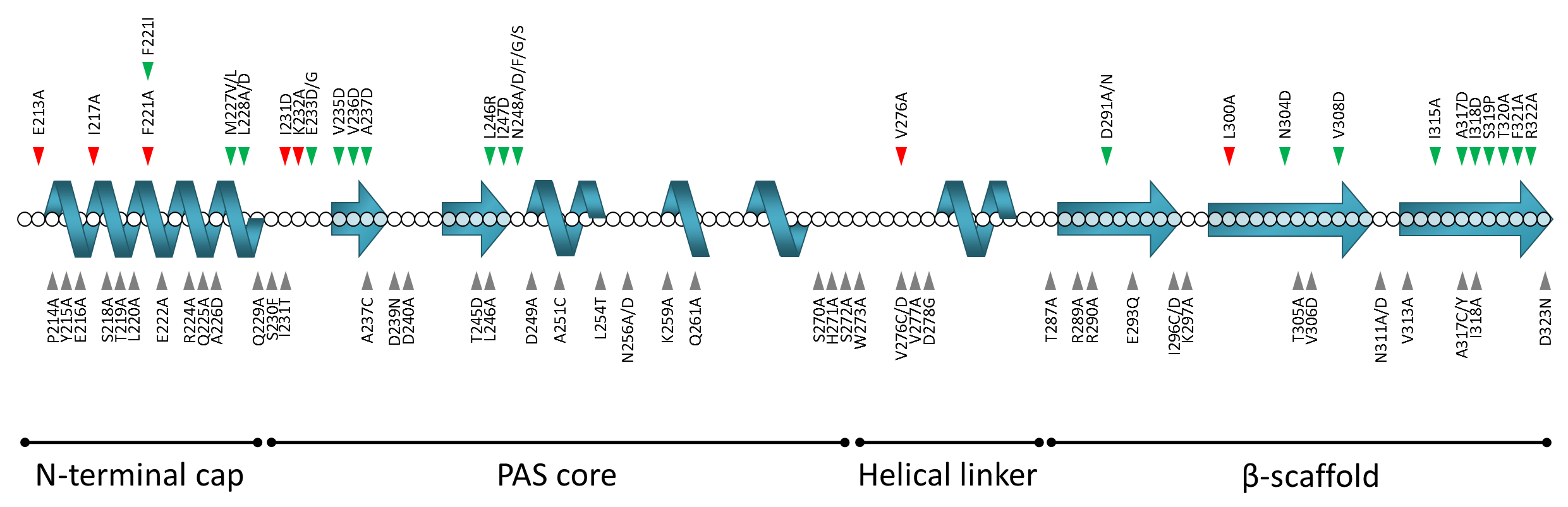
**

**Figure S4. Overview of ON (green arrows), OFF (red arrows) and silent (grey arrows) mutations in the PAS_C_ domain.**  The figure gives a summary of all ON and OFF mutations with the location and type of mutation in PAS_C_. For the definition of ON and OFF mutations see Fig. 3 and Fig. 8, respectively. See Tables 1 and S1 for the corresponding plasmids.
